# Onshore human swimming motion measurement and dynamic analysis using wearable inertial sensors

**DOI:** 10.3389/fbioe.2026.1791337

**Published:** 2026-05-07

**Authors:** Qiwei Zhang, Zijian Li, Yinxiang Bao, Hongbin Fang, Qining Wang

**Affiliations:** 1 Yiwu Research Institute, Fudan University, Yiwu, China; 2 College of Intelligent Robotics and Advanced Manufacturing, Fudan University, Shanghai, China; 3 College of Engineering, Peking University, Beijing, China

**Keywords:** inertial measurement unit (IMU), multi-rigid-body dynamic model, optical motion capture, swimming dynamics, swimming motion optimization

## Abstract

Accurately measuring and assessing human swimming performance remains challenging due to difficulties in capturing full-body motion in horizontal postures and evaluating swimming dynamics based on measured data. This study proposes an integrated framework combining wearable inertial measurement units (IMUs), an onshore swim trainer, and a multi-rigid-body dynamic model to measure swimming kinematics and evaluate swimming performance. Seventeen wireless IMUs are used to capture full-body motion data during onshore breaststroke, freestyle, and butterfly strokes, and comparison with optical motion capture data demonstrates that the IMU-based measurement scheme has good validity (Spearman’s correlation >0.75), reliability (ICC >0.75), and accuracy (NRMSE <25%) for most body segments. However, lower limb and trunk motions deviate from typical in-water patterns due to restricted downward swing on the onshore trainer. To assess swimming performance with the IMU-measured data, a Newton-Euler dynamic model incorporating fluid forces is developed. Simulations reveal that stroke frequency (SF) has a significant effect on swimming speed and propulsion force across the three strokes. Two case studies further demonstrate the framework’s potential for motion optimization: modifying arm movements in freestyle and trunk movements in butterfly can improve swimming performance. Overall, this framework enables reliable and efficient onshore swimming motion measurement, dynamic performance assessment, and individualized technique optimization, which could provide a supplementary tool and preliminary screening method for guiding swimming training and swimming robot development.

## 1 Introduction

Human swimming involves intricate and dynamic joint movements that vary greatly across different strokes. Hence, accurate assessment of swimming performance necessitates effective motion measurement and analysis methods, which are essential for both training optimization (Liu et al., 2021) or swimming robot development (Nakashima and Kuwahara, 2016; Nakashima et al., 2021). While optical-based measurements are considered the gold standard for human swimming motion capture (Ceseracciu et al., 2011; Pla et al., 2021) due to its high precision, their implementation faces several limitations and challenges. First, underwater optical motion capture systems are expensive, and the setup of waterproof optical lenses is complex and cumbersome. Second, the presence of eddies and waves stirred up by swimming motions can significantly degrade the quality of optical measurements (Callaway et al., 2009; Wang et al., 2020). The underwater environment, characterized by low lighting and occlusion from body parts, further complicates measurements, leading to loss or misinterpretation of optical marker data and increasing measurement errors. In addition, video-based data analysis is typically performed offline, which is labor-intensive and time-consuming, hindering its practicality as a real-time feedback tool (Morais et al., 2022).

In contrast to optical motion capture systems, IMUs do not require complex environment setups, such as the placement of motion capture lenses in swimming pools (Magalhaes et al., 2015; Guignard et al., 2017). Moreover, their functionality is unaffected by underwater lighting conditions, which enhances their adaptability. Certainly, it is undeniable that the accuracy of IMU measurements is lower than that of optical motion capture systems, and may suffer from issues such as signal drift. Furthermore, the devices may somewhat restrict the swimmers’ movements. However, these limitations do not outweigh their advantages, and numerous studies continue to focus on the application of IMU-based methods in swimming motion measurement, analysis, and optimization (Stamm et al., 2011; Bächlin and Tröster, 2012; Dadashi et al., 2012; 2013; Magalhaes et al., 2015; Chen et al., 2016; Zhang et al., 2017; Parvis et al., 2017; Wang et al., 2019a; 2019b; 2020; Cortesi et al., 2019; Wu et al., 2021; Guignard et al., 2021; Fantozzi et al., 2022). Existing IMU-based swimming research can be categorized into low-order and high-order parameter measurements based on the nature of the data acquired (Guignard et al., 2017). The low-order parameters are related to the common biomechanical parameters, including positions, velocities, and accelerations of the swimmer’s whole body or specific body segment. For example, Dadashi et al. (2012) mounted an IMU on the back of the swimmer to measure the forward velocity during freestyle. Bouvet et al. (2025) placed a single IMU on the sacrum of the swimmers to measure their velocity during 25-m freestyle sprints, and performed cluster analysis from both technique and performance aspects. In fact, the same testing method can also be applied to the other three swimming strokes (breaststroke, butterfly, and backstroke) (Hamidi Rad et al., 2021). Furthermore, if the IMU is mounted on specific limbs, motion data of the target limbs can be collected and analyzed. For instance, Fantozzi et al. (2022) placed IMUs on the head, feet, and other locations of the swimmer to measure the angular velocity of the corresponding body segments, while Cortesi et al. (2019) used IMUs to obtain the 3D wrist trajectory of the hand during freestyle swimming. In particular, since these studies aim to obtain the swimmer’s body segment velocity in the global coordinate system, it is necessary to process the IMU measurements and transform the data from the local coordinate system of the IMUs to the global coordinate system of the measurement environment. High-order parameters are derived from the analysis of low-order parameters, such as the relative phase between different body segments. For instance, Guignard et al. (2021) conducted both onshore 2D motion experiments and 3D arm swing experiments, comparing the results with optical motion capture data. Their findings confirmed the reliability, validity, and accuracy of IMU-based measurements of human joint angles. Additionally, they mounted IMUs on swimmers’ arms and hands to analyze breaststroke movements and the variability of angles during different motion cycles. Wang et al. (2020) utilized IMUs to collect motion data during both upright imitation of swimming movements and actual swimming in the pool. They employed supervised learning to segment the four swimming strokes into distinct phases. In our previous research, we also employed IMUs to measure the time-history data of knee angle changes, and used these data to classify different swimming strokes (Zhang et al., 2017). In another research, we used IMUs and optical motion capture system to obtain Euler angles of the lower limb, and reconstruct the underwater kinematic model (Wu et al., 2021).

Although previous studies have provided preliminary evidence supporting the feasibility of wearable IMUs for swimming motion measurement, several limitations remain in IMU-based approaches. First, much of the existing research has focused on placing IMUs on specific body segments of interest, without considering the movement of the entire body during swimming. For example, IMUs have been mounted on the lower limbs to study joint movements (Guo et al., 2024). A single IMU has also been placed on the sacrum to measure the body’s forward velocity in various swimming strokes (Hamidi Rad et al., 2021). However, it is crucial to note that effective swimming performance assessment and instruction require a comprehensive understanding of the movement patterns of all major segments throughout the body. For example, during freestyle swimming, swimmers may maintain identical upper-limb movements while exhibiting completely different lower-limb kicking frequencies, including six kicks or eight kicks per stroke cycle, which can significantly influence swimming speed (Maglischo, 2003; Gourgoulis et al., 2014). As a result, it is essential to investigate full-body data in swimming motion.

Secondly, prior studies often conducted simplified tests of swimming movements, such as waving or upright imitation, on land. The effectiveness and accuracy of IMU-based methods were then validated by comparing IMU data with optical motion capture data. However, real swimming involves the human body in a horizontal position with complex joint movements, which differ significantly from upright motions and presents additional challenges. This disparity underscores the need for further validation of IMU-based methods in swimming scenarios where the human body is horizontal. Note that in actual swimming tests conducted in water, comparative analyses of IMU data with optical motion capture data have been less frequently performed due to the high hardware demands of underwater optical motion capture system and the difficulty of collecting comprehensive data from all body segments (Dadashi et al., 2012; Guignard et al., 2021; Slopecki et al., 2024). Considering that land-based measurements impose lower requirements on sensor equipment, are easier to implement, and allow comparison with optical motion capture as the gold standard, we use a land-based measurement framework. This approach can be used to evaluate the validity, reliability, and accuracy of IMU measurements, as well as to verify the effectiveness of the dynamic simulation method. In this setup, swimmers are required to perform different swimming movements while maintaining a horizontal posture.

In addition to the measurement challenges discussed above, once human swimming data is collected, another challenge arises: how to effectively analyze and utilize the obtained data. One potential approach is to conduct swimming dynamic simulations based on the IMU data. Existing simulation methods for swimming can be broadly categorized into two types: computational fluid dynamics (CFD) modeling and multibody dynamics modeling. The advantage of the former method is that it provides relatively high computational accuracy. First, a high-precision 3D human model (e.g., a musculoskeletal model (Bixler et al., 2007; Lauer, 2023) needs to be established, along with swimming motion data, and then imported into CFD software (Takagi et al., 2016). From there, flow field parameters need to be determined to calculate swimming velocity (Zaïdi et al., 2008), fluid forces (Li and Zhan, 2015), and the propulsion generated by limb movements (Pacholak et al., 2014). Corresponding simulation results can already provide a bridge between kinematics and fluid dynamic modeling; however, it should be noted that CFD analysis also requires substantial computational cost, which poses challenges for tasks such as limb motion optimization. In contrast, multibody dynamics simulations offer greater efficiency. This method involves simplifying the human body into a multi-rigid body model and deriving limb motion patterns from experimentally measured swimming motion signals (Nakashima et al., 2007). For example, using an 18-segment rigid-body model, Nakashima et al. developed the Swimming Human Model (SWUM) (Nakashima et al., 2007) to analyze different swimming strokes and optimize stroke paths (Nakashima et al., 2018; 2020). It is worth noting that the multibody dynamic model has some limitations, including relying on pre-measured kinematic signals of joint movements, ignoring the interactive effects between limbs, and yielding less accurate fluid forces than CFD simulations. However, owing to its high computational efficiency, this module could be adopted in exploring the qualitative features of various swimming strokes, interpreting swimming dynamics, and optimizing motion patterns.

Given the three challenges outlined, research on using IMUs to capture data from all major body segments in horizontal positions across different swimming strokes is limited, and even fewer studies have compared this data with gold-standard optical motion capture data. Moreover, there is a significant need to further predict swimming dynamics and performance based on measured human swimming motion data. To address these gaps, this study employs an onshore swim trainer, which is commonly used by professional swimmers during training. Using this setup, we conducted onshore tests for breaststroke, freestyle, and butterfly stroke, collecting both optical motion capture and IMU data from all major segments during the exercises. By comparing these two datasets, we comprehensively and systematically assess the validity, reliability, and accuracy of IMUs in measuring human swimming motions. Specifically, the validity refers to the degree to which the IMUs measure what they are expected to measure; reliability describes the consistency and repeatability of measurements obtained under the same experimental conditions; accuracy is the proximity between the measured values and the reference or true values (Guignard et al., 2021). Additionally, we employ the multi-body Newton-Euler dynamics model developed in our previous work (Bao et al., 2022) as the core framework for swimming dynamic simulations. By inputting experimentally measured human segment geometry and IMU motion data, this model enables rapid computation of swimming dynamics, yielding various performance metrics such as swimming speed and propulsion force, alongside intuitive swimming simulation animations. This model also facilitates the analysis of the effects of SF and motion optimization on swimming performance across the three strokes. Overall, the swimming motion measurement scheme and the performance prediction method based on a rigid-body dynamic model proposed in this study can serve as a supplementary tool or preliminary screening method for real-water swimming training and swimming robot development, offering innovative solutions for both fields.

The rest of this article is organized as follows: Section 2 outlines the experimental setup, the IMU data processing methods, data evaluation metrics, and dynamic simulation framework. Section 3 quantitatively compares and analyzes the IMU data and optical motion capture data to comprehensively evaluate the validity, reliability, and accuracy of the IMU-based approach in swimming motion measurement. We also discuss the dynamic simulation results based on IMU data, correct and optimize the movements of different swimming strokes in simulations to achieve higher swimming speeds. Section 4 discusses the limitations of this study and highlights potential directions for future applications. Finally, Section 5 provides the conclusions and outlook.

## 2 Materials and methods

### 2.1 Measurement setup

**FIGURE 1:**
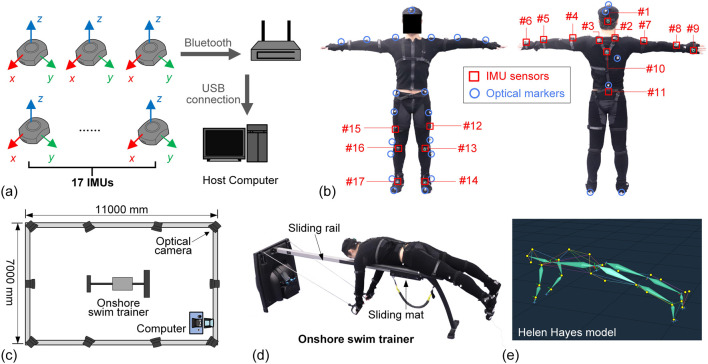
The IMU and the optical motion capture systems: **(a)** IMU coordinates and signal acquisition. **(b)** IMU and optical marker positions. **(c)** Experimental setup, where the optical motion capture system is sketched. **(d)** The photo of the athlete lying on the onshore swim trainer. **(e)** Schematic diagram of the human body based on the Helen-Hayes model.

In this study, two measurement systems are employed: inertial measurement units (IMUs) and an optical motion capture system. The IMUs used are wireless nine-axis MEMS sensors (Perception Neuron Studio, Noitom Technology Ltd., Beijing, China), which integrate a gyroscope (range: ±2000), an accelerometer (range: ±32 G), and a magnetometer (details are provided in Supplementary Material S1). Each sensor measures 43 mm × 33 mm × 20 mm and weighs only 15.8 g. The reported static errors for roll, pitch, and yaw are 0.7°, 0.7°, and 2°, respectively, with a minimum Euler angle resolution of 0.02°. The IMUs transmit data wirelessly via Bluetooth to a transceiver, which then relays the data to a host computer through a USB connection (Figure 1a). Multiple data output frame rates are supported, and a sampling rate of 60 Hz is selected in this study. During testing, 17 IMUs are securely mounted to the subject’s body and limbs using straps, as shown in Figure 1b, Table 1 (the specific placement of the IMU is listed in Supplementary Table S1). By integrating the sensor data with a pre-defined human skeletal model, the Euler angles of each limb and body segment are estimated. By integrating the sensor data with a pre-defined human skeletal model given in the Perception Neuron Studio system (details are provided in Supplementary Material S1), the Euler angles of each limb and body segment are estimated. The obtained Euler angle data have been processed by the built-in extended Kalman filter algorithm in Perception Neuron Studio, and the smoothing and filtering operations have been completed. Prior to data collection, all IMUs are initialized and calibrated to align their initial orientation with the subject’s posture and to eliminate errors arising from sensor bias or environmental disturbances. Specifically, the swimmer needs to perform two calibration poses, A-pose and T-pose (Figure 1b), following the system’s user guide. In the A-pose, the swimmer needs to stand with palms resting against the thighs and feet parallel. In the T-pose, the swimmer needs to abduct their arms to 90° with palms facing downward. In the following sections, we will establish a multi-rigid-body dynamic model and use the Euler angle data obtained from IMUs to calculate the pose data of each body segment.

**TABLE 1:** Division, corresponding nodes, indexes for validation, and IMU arrangement for 18 human body segments.

Segment	Boundary	Nodes	Indexes for validation	IMUs
Head	Top of head to lower margin of mandible	11, 18	Angles about global *X*-axis	#1
Neck	Inferior border of mandible to upper margin clavicle	10, 11	N/A	Virtual
Shoulder	Upper margin clavicle to acromion	9, 10	N/A	#2, #3
Left upper arm	Acromion to the left elbow joint	15, 16	Angles about global *X*, *Y*, *Z*-axes	#4
Left forearm	Left elbow joint to left wrist joint	16, 17	Relative angle between left upper arm and left forearm	#5
Left hand	Left wrist joint to left middle fingertip	17, 22	N/A	#6
Right upper arm	Acromion to the right elbow joint	12, 13	Angles about global *X*, *Y*, *Z*-axes	#7
Right forearm	Right elbow joint to right wrist joint	13, 14	Relative angle between right upper arm and right forearm	#8
Right hand	Right wrist joint to right middle fingertip	14, 21	N/A	#9
Upper trunk	Acromion to chest sword joint	8, 9	N/A	#10
Middle trunk	Chest sword joint to the upper umbilicus	7, 8	N/A	Virtual
Lower trunk	Upper umbilicus to anterior superior spine	0, 7	N/A	#11
Left thigh	Anterior superior spine to left knee joint	4, 5	Angles about global *X*, *Y*, *Z*-axes	#12
Left shank	Left knee joint to lower margin of left medial malleolus	5, 6	Relative angle between left thigh and left shank (left knee angle)	#13
Left foot	Lower margin of left medial malleolus to the left sole	6, 20	Angles about global *X*-axis	#14
Right thigh	Anterior superior spine to right knee joint	1, 2	Angles about global *X*, *Y*, *Z*-axes	#15
Right shank	Right knee joint to lower margin of right medial malleolus	2, 3	Relative angle between right thigh and right shank (right knee angle)	#16
Right foot	Lower margin of right medial malleolus to the right sole	3, 19	Angles about global *X*-axis	#17

An optical motion capture system (Vicon Vero) (Vicon Motion Systems Ltd., Oxford, UK) is used as the gold standard to validate the accuracy of the data collected from the IMUs. Reflective markers are attached to the subject’s body following the Helen Hayes marker set protocol (Figure 1b, Table 1), which originally includes 29 markers −13 on the trunk and upper limbs, and 16 on the lower limbs. However, due to occlusion of markers on the medial side of the lower limbs during actual tests, virtual markers are generated for the knee and ankle joints based on the kinematic relationships of the lower limbs. Hence, the number of physical optical markers is reduced to 25 (Figure 1b). As shown in the experiment setup (Figure 1c), 10 optical cameras are used to track the three-dimensional trajectories of each marker in real time. Combining these trajectories with the Helen Hayes model enables the calculation of segmental kinematics, including the Euler angles of individual limbs and body segments during swimming motions. Prior to data acquisition, the optical motion capture system is calibrated to define the measurement volume accurately and to minimize the influence of environmental noise.

To enable swimmers to simulate various strokes on land, an onshore swim trainer (VASA Swim Ergometer) (Vasa, Inc., Essex Junction, VT, United States) is employed in this study (Figure 1d). The trainer comprises a main support frame, a sliding mat, a slide rail, and an adjustable wind resistance system. The wind resistance mechanism modulates the load applied to the swimmer, thereby replicating the hydrodynamic resistance experienced in water. The resistance module has a total of seven levels available for setting, and level three is consistently used in the following experiments.

During use, the subject lies prone on the sliding mat, with both hands wearing hand paddles connected to the wind resistance system. As the swimmer performs stroke motions, the tension generated by the resistance system propels the sliding mat forward and backward along the rail, effectively simulating the posture and dynamics of real swimming. Figure 1e illustrates a geometric model of the subject in a horizontal position on the trainer, constructed using 25 markers based on the Helen-Hayes protocol. Note that this type of equipment is widely adopted by professional swim teams for regular training, allowing swimmers to perform proper strokes on the machine.

### 2.2 Methods

The core concept of the proposed motion measurement method is to estimate the subject’s body posture using IMUs. Specifically, the IMUs capture gyroscope data, which is used to compute Euler angles and derive the corresponding rotation matrices. In parallel, the optical motion capture system directly records the spatial positions of reflective markers. Combined with anthropometric data, this positional information allows for the calculation of limb motion Euler angles. After verifying the IMU-based method in terms of its validity, reliability, and accuracy for measuring human swimming movements, the acquired motion data is further used for performing a multi-rigid-body dynamics simulation. This enables quantitative evaluation of swimming performance (i.e., velocity and propulsive force) and dynamics as well as generating the animation videos the swimmer’s motion.

#### 2.2.1 System coordinate definition and rotation matrix

**FIGURE 2:**
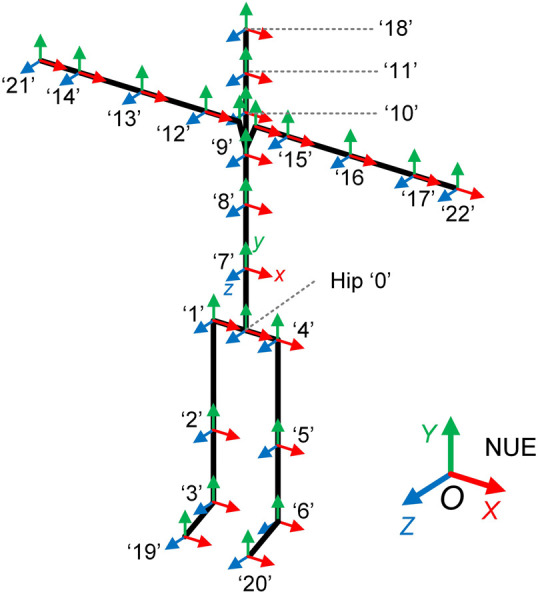
Coordinate system of each IMU at the initial static calibration position.

Prior to measurement, each IMU must be properly positioned. The sensor should lie flat on the surface of the corresponding limb segment, with its local coordinate frame aligned as closely as possible with the distal joint coordinate frame (Wu et al., 2002). To compute the Euler angles for each segment, it is necessary to define the direction and order of Euler angle rotations. The initial static coordinate systems of all IMUs are aligned to the global NUE (North-Up-East) frame {G}, as shown in Figure 2. A total of 17 IMUs are used, covering major body segments including head, neck, shoulders, left and right upper arms, forearms, and hands; upper, middle, and lower trunk; left and right thighs, calves, and feet (see Table 1).

For each IMU, angular velocity data acquired from the gyroscope, accelerometer, and magnetometer is used to compute the corresponding Euler angles that describe the orientation of the limb, and the associated rotation matrices are generated for use in subsequent multi-rigid-body dynamics simulations. In this paper, the rotation conventions are defined as follows: rotation about the *x*-axis is specified as yaw ( ϕ ), rotation about the *y*-axis as pitch ( ψ ), and rotation about the *z*-axis as roll ( θ ). The Euler angles ( ϕ , ψ , θ ) of each IMU represent the orientation of the IMU’s local coordinate system relative to the global reference frame *O-XYZ*. Specifically, the transformation is achieved by sequentially rotating the initial coordinate system: first around the *z*-axis by θ , then around the *x*-axis by ϕ , and finally around the *y*-axis by ψ . The associated rotation matrix yields

$$R_{z}\left(\theta \right)=\left[\begin{array}{ccc} \cos\theta & -\sin\theta & 0\\ \sin\theta & \cos\theta & 0\\ 0 & 0 & 1 \end{array}\right],$$ (1)

$$R_{x}\left(\phi \right)=\left[\begin{array}{ccc} 1 & 0 & 0\\ 0 & \cos\phi & -\sin\phi \\ 0 & \sin\phi & \cos\phi \end{array}\right],$$ (2)

$$R_{y}\left(\psi \right)=\left[\begin{array}{ccc} \cos\psi & 0 & \sin\psi \\ 0 & 1 & 0\\ -\sin\psi & 0 & \cos\psi \end{array}\right].$$ (3)

Hence, the attitude matrix of the corresponding limb can be calculated by

$$R=R_{z}\left(\theta \right)R_{x}\left(\phi \right)R_{y}\left(\psi \right).$$ (4)

Since human body size significantly influences motion measurement results, a generic human joint coordinate system template is developed to standardize the analysis. The human body model consists of 17 segments and 23 nodes, which are sequentially numbered from ‘0’ to ‘22’ (as shown in Figure 2; Table 1). The hip joint is designated as the root node, and the local coordinate system of its corresponding IMU is aligned with the global reference frame. Using the rotation matrix obtained from the IMU data, the global positions of all nodes can be calculated using the following equation,

Pfnf0=∑i=1n−1∏j=1iRfjfj−1vfi→fi+1fi+P0f0, (5)

enabling a complete reconstruction of the subject’s posture during motion. Here, f denotes the vector containing the indices of all nodes along the kinematic chain from a target node to the root node. For example, to compute the global position of node ‘18’, $f=\left[0,7,8,9,10,11,18\right]$ ), and n represents the length of f . The vector vfi→fi+1fi denotes the vector in the local frame { $f\left(i\right)$ }, pointing from node $f\left(i\right)$ to node $f\left(i+1\right)$ when the subject is in an upright calibration posture. The length of this vector is determined by the measured distance between adjacent joints of the subject. Note that the coordinate frame { $f\left(0\right)$ } corresponds to the global reference frame {G}. Rfjfj−1 is the rotation associated with the segment connecting node $f\left(j\right)$ to $f\left(j-1\right)$ is calculated based on the IMU-derived attitude matrix using Equation 4. The global position of the root node ‘Hip’ is defined as P0f0=0,h,0 , where h is the measured distance from the midpoint of the hip joint to the base of the feet.

Note that the neck and middle trunk are modeled as virtual segments without directly attached IMUs (see Table 1). The lengths of these segments are determined by measurement, while their orientations are estimated based on adjacent segment kinematics. Specifically, the Euler angles of the neck are approximated as the average of angles measured at the shoulders and the head, and those of the middle trunk are estimated as the average of the upper and lower trunk Euler angles. Using this skeletal model and the corresponding transformation matrices, the global positions of all joints can be computed in the NUE reference frame.

#### 2.2.2 Experimental process

Based on the experiment setup shown in Figure 1, the subject’s swimming motion data are simultaneously recorded using both the IMU system and the optical motion capture system. Due to the limited visibility of optical markers placed on the subject’s back during backstroke, reliable data acquisition for this stroke is not feasible. Therefore, this study focuses exclusively on breaststroke, freestyle, and butterfly strokes.

The stroke frequencies for each swimming style are listed in Table 2. For each combination of stroke type and stroke frequency, kinematic measurements were conducted over a continuous period of at least 45 s to ensure sufficient data for analysis. To minimize fatigue and ensure consistent performance, the subject was given a 3-min rest interval between different stroke frequencies of the same swimming stroke and a 30-min rest period between tests involving different swimming strokes.

**TABLE 2:** Experimental protocol for human swimming measurements.

Swimming stroke	Duration (s)	Stroke frequency (stroke per minute, SPM)	Rest time (min)
Breaststroke	45	30,40,50	3
30 min break			
Freestyle	45	60, 80,100	3
30 min break			
Butterfly stroke	45	30,40,50	3

#### 2.2.3 Signal processing

During the measurement process, both the IMU and optical motion capture systems independently record kinematic data; however, discrepancies in their respective start times result in a time offset between the two datasets. To ensure accurate comparison, it is essential to temporally align the signals by compensating for this delay. Given that the time shift between the systems is constant across all segments for a given swimming stroke and frequency, alignment can be achieved by referencing the motion of a single representative body segment. In this study, the left arm is selected as the reference segment due to its pronounced periodic motion during swimming.

Specifically, the time corresponding to the first peak in the optical motion capture data for the left arm is identified and used as the temporal reference point. The IMU data is then time-shifted such that the first peak in the IMU signal aligns with the reference point. Following this temporal alignment, the IMU data are further adjusted to ensure that the initial angle values obtained from the IMU system align with those recorded by the optical motion capture system, thereby achieving both temporal and initial value synchronization between the two datasets.

These alignment procedures are subsequently applied uniformly to all IMU data. The validity, accuracy, and reliability of IMU-based measurements are then assessed based on the aligned datasets, enabling direct comparison with the gold-standard optical motion capture system.

#### 2.2.4 Validity, reliability, and accuracy indexes

##### 2.2.4.1 Validity indexes

Since the measured human joint angles do not satisfy the assumption of normality, the Spearman rank correlation coefficient is used to evaluate the strength and direction of the monotonic relationship between the IMU data and the optical motion capture data. Unlike Pearson’s correlation, Spearman’s correlation does not require normally distributed data and is suitable for evaluating non-linear but monotonic associations. The Spearman correlation coefficient ranges from +1, indicating a perfect positive monotonic correlation, to −1, indicating a perfect negative monotonic correlation. The Spearman correlation coefficient between the IMU data and motion capture data is calculated by (Puth et al., 2015)

$${\text{Spearman}}^{\prime }r=\frac{\sum_{i=1}^{n}\left({A_{\text{IMU},}}_{i}-{\bar{A}}_{\text{IMU}}\right)\left({A_{\text{Opt},}}_{i}-{\bar{A}}_{\text{Opt}}\right)}{\sqrt{\sum_{i=1}^{n}{\left({A_{\text{IMU},}}_{i}-{\bar{A}}_{\text{IMU}}\right)}^{2}\sum_{i=1}^{n}{\left({A_{\text{Opt},}}_{i}-{\bar{A}}_{\text{Opt}}\right)}^{2}}}.$$ (6)

Here, ${A_{\text{IMU},}}_{i}$ and ${A_{\text{Opt},}}_{i}$ represent the angles measured at the *i*th time point by the IMU and the optical system, respectively, while ${\bar{A}}_{\text{IMU}}$ and ${\bar{A}}_{\text{Opt}}$ denote their corresponding average values over the entire dataset. n denotes the total number of time-series data points. A Spearman correlation coefficient close to 1 indicates a strong positive monotonic correlation between the IMU and optical motion measurements, implying high consistency in their temporal trends. In contrast, a coefficient closer to 0 suggests a weaker or no monotonic correlation between the two datasets, suggesting lower agreement in the temporal profiles of the measured angles.

In addition, linear regression analysis is performed to further evaluate the relationship between the IMU measurements and optical motion capture data. The coefficient of determination $R^{2}$ is used as a metric to quantify the goodness of fit. An $R^{2}$ value closer to 1 indicates a high level of consistency and agreement between the two datasets, signifying that the IMU measurements closely approximate those obtained from the gold-standard optical system.

##### 2.2.4.2 Reliability indexes

To assess the consistency between the two measurement systems, the Absolute Agreement Intraclass Correlation Coefficient (ICC) is evaluated using a one-way random effects model (Koo and Li, 2016). The ICC quantifies the degree of absolute agreement between the two measurement methods across all data points. The ICC in a one-way random effects model yields:

$$\begin{array}{l} \text{ICC}\left(1,1\right)=\frac{{\text{MS}}_{R}-{\text{MS}}_{W}}{{\text{MS}}_{R}+\left(k-1\right){\text{MS}}_{W}},\\ {\text{MS}}_{R}=D\left(\frac{A_{\text{IMU}}+A_{\text{Opt}}}{2}\right)\times 2,\\ {\text{MS}}_{W}=\left(\sum_{i=1}^{n}D\left({A_{\text{IMU},}}_{i},{A_{\text{Opt},}}_{i}\right)\right)/n. \end{array}$$ (7)

Here, $A_{\text{IMU}}$ and $A_{\text{Opt}}$ represent the data vectors obtained from the IMU and optical motion capture measurements, respectively. The operator $D\left(\cdot \right)$ denotes the calculation of variance. ${\text{MS}}_{R}$ represents the mean square for rows (i.e., between-subset variance), and ${\text{MS}}_{W}$ denotes the mean square of the residuals (i.e., within-subject or error variance). *k* is the number of measurement methods (here, *k* = 2 for IMU and optical motion capture), and *n* is the total number of data points. In general, an ICC value approaching 1 indicates excellent reliability and strong agreement between the IMU and optical motion capture data, while a value approaching 0 reflects poor reliability and significant inconsistency between the two measurement systems.

##### 2.2.4.3 Accuracy indexes

The normalized root mean square error (NRMSE) is a commonly used normalized measure of the difference between the predicted values and the corresponding reference (true) values. It provides an overall measure of the deviation across all data points and is particularly useful for evaluating the precision of measurement systems. Therefore, in this study, the NRMSE between the IMU-derived angle data and the corresponding optical motion capture data is calculated to assess the accuracy of the IMU-based measurement system. The NRMSE is computed as follows:

$$\text{NRMSE}=\frac{\sqrt{\frac{1}{n}\sum_{i=1}^{n}{\left(A_{\text{IMU},i}-A_{\text{OPT},i}\right)}^{2}}}{\left(\max\left(A_{\text{Opt}}\right)-\min\left(A_{\text{Opt}}\right)\right)}.$$ (8)

A lower NRMSE value indicates higher accuracy and better agreement between the two datasets.

#### 2.2.5 Body 3D scanning and sizing

To accurately determine the geometric parameters of the human body required for dynamic modeling, a three-dimensional body scan of the subject was conducted using a multifunctional handheld 3D scanner (EinScan Pro 2X Plus, Shining 3D). The subject was an adult male university student (age: 26 years; height: 1.71 m; weight: 65.0 kg, a National Master of Sports (swimming) with 22 years of systematic swimming training). The experimental protocol was approved by the Ethical Committee of Fudan University (protocol code: FE21124, approved date: 16 August 2021). Prior to participation, the subject was fully informed of the study objectives and procedures and provided a written informed consent form.

**FIGURE 3:**
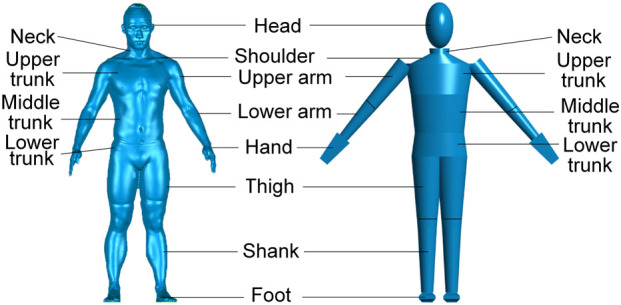
Geometric modeling of the human body. The left subfigure shows the reconstructed 3D model of the subject’s body obtained via 3D scanning. The right subfigure displays the corresponding multi-rigid-body model consisting of 18 segments.

As shown in Figure 3, the point cloud data of the subject’s body was obtained via the 3D scan, and a detailed 3D CAD model of the subject’s human body was subsequently reconstructed. To facilitate dynamic simulation in later sections, the anatomical 3D model was simplified into a multi-rigid-body model. Based on morphological features, body segments were geometrically approximated using three canonical shapes: ellipsoid, truncated elliptical cone, and frustum of a pyramid. The characteristic dimensions (e.g., length, major/minor axes, and diameters) of each human-body segment were measured directly from the 3D model and mapped to the corresponding rigid-body segment in the multi-rigid-body model, as detailed in Table 3.

**TABLE 3:** Geometric parameters and density of model segments.

Segment	Geometric shape	Geometric Parameters (cm)					Density (kg/m^3^)
Head	Ellipsoid	dsl		Semi-minor axis		Axial length	​
		20.73		16.24		26.68	1,042
​	Truncated elliptical cone	Semi-axis R_1_ ^a)^	Semi-axis R_2_ ^b)^	Semi-axis T_1_ ^c)^	Semi-axis T_2_ ^d)^	Axial length	​
Neck		13.87	11.13	12.87	11.78	3.09	1,042
Shoulder		19.75	39.56	13.87	11.13	5.75	1,042
Upper trunk		24.35	33.38	19.75	39.56	17.81	700
Middle trunk		21.63	28.60	24.35	33.38	22.24	1,042
Lower trunk		21.63	28.60	21.17	32.41	13.81	1,042
Upper arm		10.33	10.33	8.36	8.36	30.14	1,042
Forearm		8.36	8.36	4.71	5.67	25.86	1,042
Thigh		18.54	18.22	12.15	11.11	41.33	1,042
Shank		12.15	11.11	9.83	7.06	37.40	1,042
​	Frustum of a pyramid	Side length R_1_ ^a^	Side length R_2_ ^b^	Side length T_1_ ^c^	Side length T_2_ ^d^	Axial length	​
Hand		4.64	10.13	1.40	5.24	19.80	1,042
Foot		5.45	6.94	1.86	10.06	25.03	1,042

^a^ R_1_: means the sagittal axis of the section at the root of the segment.

^b^ R_2_: means the coronal axis of the section at the root of the segment.

^c^ T_1_: means the sagittal axis of the section at the tip of the segment.

^d^ T_2_: means the coronal axis of the section at the tip of the segment.

For modeling convenience, geometric symmetry was assumed between left and right body segments, although minor asymmetries exist in the actual human body. This assumption is considered reasonable and would not significantly affect the simulation results. To enhance the realism of the dynamic analysis, the density of each body segment is uniformly set to 1,042 kg/m^3^ (except for the upper trunk, whose density was set to 700 kg/m^3^), based on our prior research (Bao et al., 2022), ensuring that the total mass of the multi-rigid-body model closely matches the subject’s actual body weight.

#### 2.2.6 Dynamic model of human swimming

Previous research has predominantly focused on the measurement and analysis of human swimming motion using various sensing technologies. A key advancement in this study is the integration of IMU-based swimming motion data with dynamic simulation, enabling the evaluation of swimming efficiency and velocity beyond mere kinematic analysis. To this end, a human swimming dynamics model is developed based on the multi-rigid-body representation of the human body constructed above. This model allows for quantitative prediction of swimming performance metrics by simulating limb movements and their associated dynamic responses.

**FIGURE 4:**
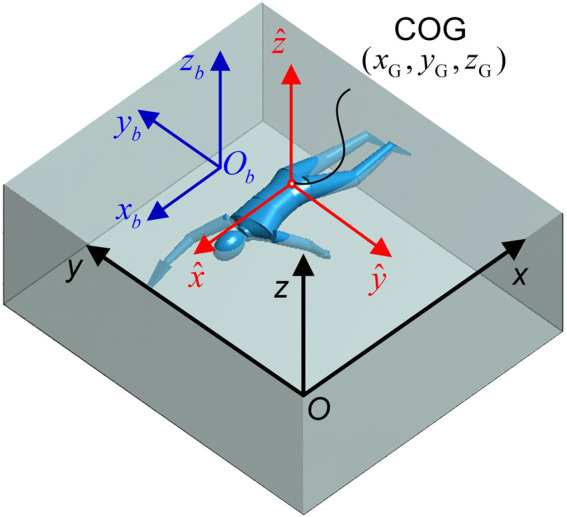
Human swimming dynamic model. Three coordinate systems are established: the global coordinate system *O-xyz*, the human body coordinate system *O*_*b*_ *-x*_*b*_ *y*_*b*_ *z*_*b*_ (the origin *O*_*b*_ locates at the root section center of the lower trunk), and the COG-principal-axes coordinate system $G-\hat{x}\hat{y}\hat{z}$ .

The dynamic process of human swimming can be described using the Newton-Euler equations of motion (Figure 4). Specifically, by modeling the human body as rigid bodies, the translational motion in the global coordinate system O−xyz can be determined according to Newton’s second law:

$$m_{\text{sub}}{\ddot{x}}_{G}=F,$$ (9)

where $m_{\text{sub}}$ represents the mass of the subject. $x_{G}={\left(x_{G},y_{G},z_{G}\right)}^{T}$ represents the position vector of the subject’s center of gravity (G) in the global coordinate system, F denotes the resultant external force acting on the human body, which includes the gravitational force, the normal and tangential drag force, the added mass force, and the buoyancy applied on the human body (Bao et al., 2022).

For a self-propulsive swimmer, the active drag is evaluated, which consists of three components: tangential drag (i.e., passive drag), normal drag, and added mass force. Each of these hydrodynamic forces is computed as the vector sum of the respective forces acting on all segments of the multi-rigid-body model. To compute these forces, each body segment is discretized into thin plates along its longitudinal axis. The fluid forces acting on each plate are assumed to be concentrated at its geometric center and are determined based on the plate’s orientation, linear velocity and acceleration, angular velocity and acceleration relative to the surrounding fluid. Buoyancy is also calculated using the differential element method, wherein the buoyant force acting on a given plate can be calculated by integrating the hydraulic pressure force over the lateral surface of a plate. For detailed formulations and derivations of these hydrodynamic force calculations, readers are referred to our previous work (Bao et al., 2022).

The rotation motion of the human body during swimming can be described by the Euler equations in the COG-principle-axes coordinate system $G-\hat{x}\hat{y}\hat{z}$

$$\left\{\begin{array}{l} J_{\hat{x}}{\dot{\omega }}_{\hat{x}}+\left(J_{\hat{z}}-J_{\hat{y}}\right)\omega_{\hat{y}}\omega_{\hat{z}}+{\dot{J}}_{\hat{x}}\omega_{\hat{x}}=M_{\hat{x}}\\ J_{\hat{y}}{\dot{\omega }}_{\hat{y}}+\left(J_{\hat{x}}-J_{\hat{z}}\right)\omega_{\hat{x}}\omega_{\hat{z}}+{\dot{J}}_{\hat{y}}\omega_{\hat{y}}=M_{\hat{y}}\\ J_{\hat{z}}{\dot{\omega }}_{\hat{z}}+\left(J_{\hat{y}}-J_{\hat{x}}\right)\omega_{\hat{x}}\omega_{\hat{y}}+{\dot{J}}_{\hat{z}}\omega_{\hat{z}}=M_{\hat{z}} \end{array}\right.,$$ (10)

where $\hat{x}$ , $\hat{y}$ , $\hat{z}$ denote the three principal axes of inertia of the human body, respectively. $J_{i}$ and $\omega_{i}$ ( $i=\hat{x},\hat{y},\hat{z}$ ) represent the rotational moment of inertia and angular velocity of the human body about the *i*-axis, respectively, while $M_{i}$ indicates the total external moment about the same axis. Due to the relative motion among body segments during swimming, the overall moment of inertia about each principal axis is time-varying. This variability necessitates consideration of the derivative of the rotational moment of inertia, which appears at the third term on the left side of Equation 10. This term captures the dynamic redistribution of mass caused by limb movements, contributing significantly to the body’s rotational dynamics during swimming.

By translating all fluid forces acting on individual body segments to the COG of the human body and computing their vector sum, the resultant external force vector F , appearing on the right-hand side of Equation 10, can be obtained. Meanwhile, by calculating the couple moments arising from force relocation and summing these contributions through appropriate vector decomposition, the external moment vector $M_{i}$ ( $i=\hat{x},\hat{y},\hat{z}$ ) on the right-hand side of Equation 10 can be determined.

#### 2.2.7 Dynamic simulation of human swimming

Based on the established dynamic model of human swimming and the IMU-derived swimming motion data, numerical simulations can be performed following the procedures listed in Algorithm 1. Simulation requires the entry of the following parameters: (1) the geometry and density of human body segments, and (2) the sequence of rotation matrices for each segment over a complete stroke cycle.

Algorithm 1. **Dynamic simulation of human swimming with IMU-based swimming motion data.**

**Input:**
1:  Segment geometry and density
2:  Relative body motion (rotational matrices) in $O_{b}-x_{b}y_{b}z_{b}$ , calculated from IMU-recorded Euler angle data
3:  Calculate the kinematics of human swimming in a cycle in $O_{b}-x_{b}y_{b}z_{b}$ (i.e., the position of the COG, the three principal axes of inertia, the principal moments of inertia)
**Set:** The number of swimming cycles $N_{\text{cycle}}$ ; the fluid field information
**Initialization:** Set the initial position and velocity of the COG, the initial orientation of the principal axes of inertia, and the initial angular velocity around the principal axes of inertia in *O*-*xyz.*
**For** *k* = 1 to $N_{\text{cycle}}$ :
1:  Calculate the absolute motion of segments in *O-xyz*;
2:  Calculate the external force **F** in *O*-*xyz* and the external torques $M_{i}$ in $G-\hat{x}\hat{y}\hat{z}$ ;
3:  Solve Equations 10, 11 via the 4th-order Runge-Kutta method to update the position and velocity of the body in *O-xyz* and the angular velocities.
**Output:** Swimming speed *v*, propulsion force **F**, and swimming simulation video.

Swimming is inherently a cyclical motion. Prior to calculating the rotation matrices, the time-series Euler angle data acquired from the IMUs must be preprocessed to obtain a representative single-cycle dataset. In dynamic simulation, the motion signals of the left and right sides of the body should be synchronized (or phase-synchronized in the case of freestyle). To achieve this, we utilize the signal from the side that exhibits higher signal quality. This selected signal is then processed through cycle averaging and interpolation (with 1000 points per cycle) to generate a smooth, continuous, single-cycle signal. The resulting signal is assigned to both legs—synchronously for breaststroke and butterfly, and with appropriate temporal translation for freestyle—to ensure symmetry (or phase synchrony) during dynamic simulations. Following this preprocessing, the rotation matrix sequence for each segment over a stroke cycle is calculated using Equations 1–4.

Using the rotation matrices computed for each segment, the relative motion of all segments over one stroke cycle can be determined. From this information, the position of the COG, the orientation of the principal axes of inertia, and the principal moments of inertia throughout the cycle can be calculated, all within the human body coordinate system $O_{b}-x_{b}y_{b}z_{b}$ . These parameters serve as essential inputs to the dynamic simulation.

To initiate the simulation, it is necessary to specify the initial conditions, which include: (1) the initial position and velocity of the COG, (2) the orientation of the principal axes of inertia, and (3) the initial angular velocities about these principal axes. These initial values define the initial configuration of $G-\hat{x}\hat{y}\hat{z}$ in O−xyz .

Following initialization and specification of the fluid field information (still water in this study), the position, velocity, and acceleration of each segment in O−xyz can be calculated. Using these kinematic quantities, the fluidic forces (in O−xyz ) and external torques (about the principal axes of inertia) acting on each segment can be determined.

Finally, the equations of motion, namely, Equations 9, 10, are numerically integrated using the 4th-order Runge-Kutta method to update the state variables—position, velocity, orientation, and angular velocity—in the global coordinate system O−xyz for the next time step.

In this study, 20 swimming cycles are simulated to ensure that the results converge to a steady state, defined by a difference in displacement and body orientation between successive cycles of less than 0.001 m and 0.01°, respectively). Once convergence is achieved, two key indicators are computed to evaluate swimming performance: (1) the swimming velocity v , which is the average forward velocity of the human body in the *x*-direction over one stroke cycle; (2) the propulsion force: the *x*-component of the external force vector F acting on the human body, which represents the net propulsive force (PF) generated during swimming.

## 3 Results

### 3.1 Evaluation of measurement and error analysis

#### 3.1.1 Validity, reliability, and accuracy evaluation

Given the nature of human swimming, the movements of the left and right body segments are approximately symmetrical (or phase symmetrical) during each stroke cycle. Therefore, the analysis of validity, reliability, and accuracy indexes in this section is restricted to the right side of the body for simplicity and consistency. Specifically, three representative stroke conditions are selected: breaststroke at 40 SPM, freestyle at 80 SPM, and butterfly at 50 SPM. For each stroke condition, the angles formed between the right upper arm, right thigh, and right shank with respect to the *x*-axis are demonstrated and analyzed. A 10-s segment of data is selected after steady-state motion is reached, and this data is used directly for evaluation without any averaging. The selected results, including Spearman’s correlation coefficient, coefficient of determination (R^2^) for linear regression, intraclass correlation coefficient (ICC(1,1)), and normalized root mean square error (NRMSE), are calculated via Equations 6–8 (summarized in Table 4) and discussed as follows. Comprehensive results for all body segments are provided in Supplementary Table S3.

**TABLE 4:** Validity, reliability, and accuracy evaluation of the IMU-based measurement approach for the three swimming strokes.

Segments	Index	Breaststroke	Freestyle	Butterfly
Right upper arm (with *x*-axis)	Spearman’s *r*	0.961	0.982	0.992
	*R* ^2^	0.916	0.967	0.990
	*ICC* (1,1)	0.948	0.979	0.994
	NRMSE (%)	10.567	6.537	3.834
Right shank (with *x*-axis)	Spearman’s *r*	0.853	0.968	0.945
	*R* ^2^	0.931	0.901	0.863
	*ICC* (1,1)	0.963	0.893	0.855
	NRMSE (%)	9.138	15.651	20.799
Right thigh (with *x*-axis)	Spearman’s *r*	0.875	0.615	0.915
	*R* ^2^	0.856	0.391	0.814
	*ICC* (1,1)	0.840	0.119	0.420
	RRMSE (%)	12.754	29.973	27.074

**FIGURE 5:**
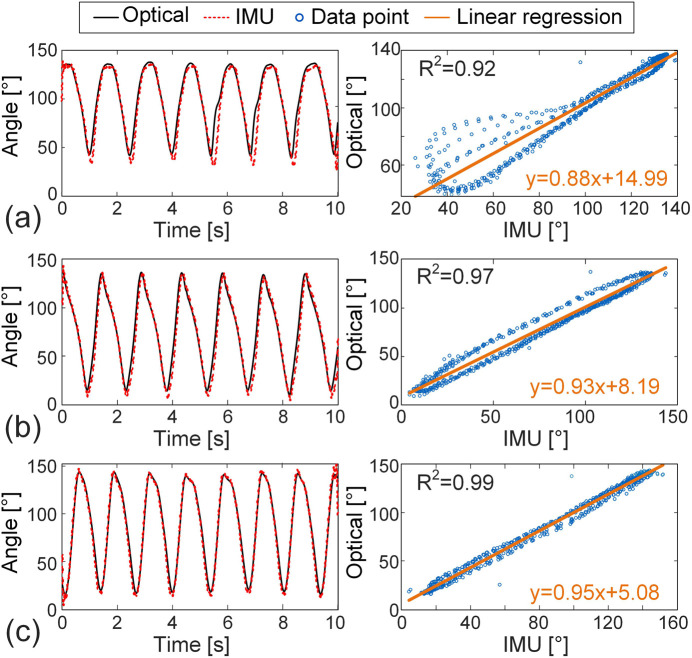
Comparison of the IMU-derived and optical motion capture results for the angle between the right upper arm and the *x*-axis across three distinct strokes (left column) and the corresponding linear regression plots (right column). **(a)** Breaststroke, **(b)** freestyle, **(c)** butterfly.

Figure 5 presents a comparative analysis of the IMU-derived and optical motion capture results for the angle between the right upper arm and the *x*-axis across three distinct swimming strokes. The time-series data indicate that the motion of the right upper arm exhibits clear periodicity and high consistency between the two measurement systems. Quantitatively, the Spearman’s *r* for all three strokes exceeds 0.96, and the R^2^ value for the linear regression approach 1.0, indicating strong monotonic and linear agreement between the IMU and gold-standard optical measurements. Moreover, ICC (1,1) ranges from 0.948 to 0.994, reflecting excellent measurement reliability. The NRMSE values are all below 11%, further confirming the accuracy of the IMU-based angle estimation. These results collectively validate the effectiveness of IMUs for accurately capturing upper limb kinematics during swimming.

**FIGURE 6:**
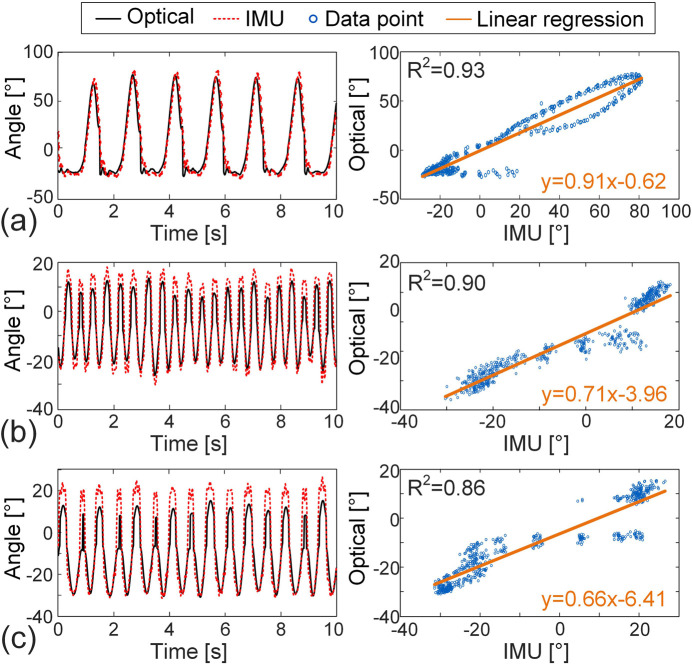
Comparison of the IMU-derived and optical motion capture results for the angle between the right shank and the *x*-axis across three distinct strokes (left column) and the corresponding linear regression plots (right column). **(a)** Breaststroke, **(b)** freestyle, **(c)** butterfly.

Figure 6 shows the comparison of right shank angles measured by IMU and optical motion capture across the three strokes. The time-series data exhibit clear periodicity in all cases. The Spearman’s *r* for breaststroke, freestyle, and butterfly are 0.853, 0.968, and 0.945, respectively, while the R^2^ values from linear regression exceed 0.86, confirming the validity of IMU measurement. The ICC (1,1) values for freestyle (0.893) and butterfly (0.855) are slightly lower than for breaststroke (0.963), yet all remain above 0.85, suggesting good reliability. The accuracy of freestyle (NRSME = 15.651%) and butterfly (NRSME = 20.799%) is a little lower than that of breaststroke (NRSME = 9.138%) due to some discrepancies in the peak-to-peak values between the IMU and optical data.

**FIGURE 7:**
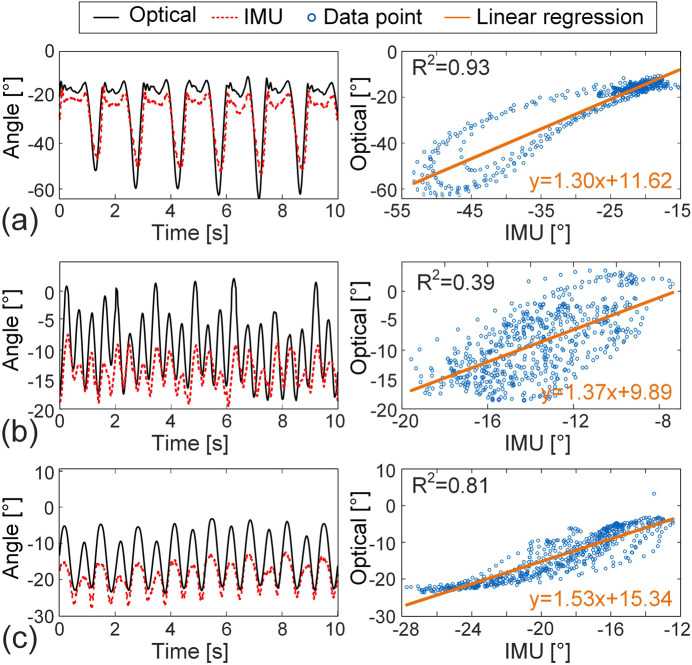
Comparison of the IMU-derived and optical motion capture results for the angle between the right thigh and the *x*-axis across three distinct strokes (left column) and the corresponding linear regression plots (right column). **(a)** Breaststroke, **(b)** freestyle, **(c)** butterfly.

For the right thigh motion (Figure 7), the IMU data for breaststroke demonstrate good validity (Spearman’s *r* = 0.875), reliability (ICC(1,1) = 0.84), and accuracy (NRSME = 12.754%). However, in freestyle, the IMU performance declines markedly, with a Spearman’s *r* of 0.615, R^2^ of 0.391, ICC(1,1) of 0.119, and NRMSE of 29.973%, indicating poor validity, reliability, and accuracy. Similarly, for butterfly, the ICC(1,1) for thigh is only 0.42, suggesting low reliability. While the overall trend of IMU and optical data in butterfly appears similar (Figure 7c), the absolute differences are substantial, underscoring the limitations of IMU-based thigh motion capture in this stroke.

Overall, the IMU-based measurement scheme demonstrates good validity, reliability, and accuracy for most body segments throughout the body in the three swimming strokes. Specifically, we evaluated 21 angles for each stroke, or a total of 63 angles, among which, 47 angles met the conditions for validity (Spearman’s correlation >0.75), 41 for reliability (ICC >0.75), and 49 for accuracy (NRMSE <25%). However, we observed that the errors in lower-limb joint angles during freestyle and butterfly are significant. Specifically, the subject’s thighs rested on the mat of the onshore swim trainer, causing the thigh muscles to undergo pronounced deformation during leg swinging. Since the IMUs are mounted at the midpoints of the thigh and shank segments, muscle deformation leads to obvious sensor displacement, which in turn affects the estimation of segment angles. In contrast, the optical motion capture markers are mainly placed near the joints, making them less affected by muscle deformation. This resulted in relatively large differences between the angles calculated from IMU data and those obtained from optical motion capture. In fact, previous studies have already reported that muscle deformation can affect the measurement accuracy of IMUs. For example, Guignard et al. (2021) observed discrepancies between IMU and optical data caused by soft-tissue artifacts when analyzing upper-limb motion in freestyle swimming, and therefore proposed mounting IMUs on rigid plates equipped with clusters of optical markers to mitigate this error. This soft-tissue artifact issue is less pronounced during real swimming in water; however, on an onshore swim trainer, the significant compression and deformation of the thigh muscles can significantly affect the calculation of kinematic angles.

#### 3.1.2 Error analysis and data correction for dynamic simulation

To investigate the sources of errors in thigh angle measurement during freestyle and butterfly strokes, we conduct a detailed analysis of video recordings. Moreover, we compare our single-cycle data (processed via cycle averaging) with the swimming motion data reported by Nakashima et al. (2007), who measured human motion data during real-water swimming using optical approaches. By inputting their lower-limb segment Euler angles into Equation 5, and adopting the geometric parameters of the subject’s 3D model based on Table 3, we can calculate the corresponding angles between each segment and the axes of the global coordinate system. Considering the difference in stroke frequency between the two datasets, temporal normalization is applied to all data. In addition, the video recordings during the experiment can also serve as an informal qualitative assessment to support the analysis of the causes of these errors.

**FIGURE 8:**
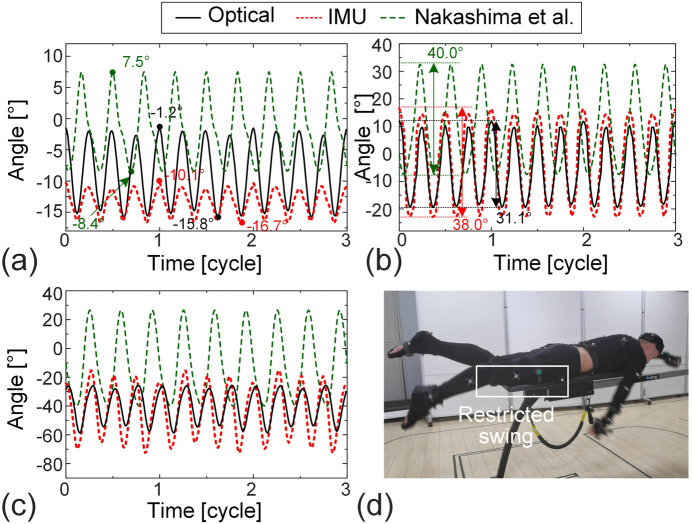
Error analysis and data correction for lower limb motions during freestyle swimming. **(a)** Thigh, **(b)** shank, and **(c)** foot. IMU data, optical motion capture data, and the data reported in Nakashima et al. (2007) are included. **(d)** The snapshot from the test video, shows the restricted motion of the thigh during tests.

Figure 8 presents the analysis results for freestyle. It should be noted that in Nakashima’s study, each stroke cycle includes three kick motions, whereas in our study, one cycle includes four kicks—reflecting individual swimming habits. Significant differences are observed in the amplitude and range of angles for the thigh (Figure 8a), shank (Figure 8b), and foot (Figure 8c). For example, the angle range of the thigh segment is $\left[-1.2^{\circ },-15.8^{\circ }\right]$ (optical) and $\left[-10.1^{\circ },-16.7^{\circ }\right]$ (IMU), where Nakashima’s data shows a markedly different range of $\left[7.5^{\circ },-8.4^{\circ }\right]$ (Figure 8a). Similarly, the amplitude of the shank segment is 31.1° (optical) and 38.8° (IMU) in our data, compared to a larger 40.0° amplitude in (Nakashima et al., 2007) (Figure 8b).

These differences are attributed to limitations inherent in our onshore test setup. Video analysis reveals that although the subject was able to perform breaststroke, freestyle, and butterfly strokes using the onshore swim trainer, the freestyle stroke—characterized by significant upward and downward swing of the lower extremities—is constrained. Specifically, the sliding mat of the swim trainer impedes the downward swing of the thighs (Figure 8d), which in turn restricts the amplitude and range of the shank and foot movements. Secondly, due to the absence of buoyancy, muscle activation in the human body differs from that in actual aquatic swimming, which consequently affects limb movements. As a result, to improve the fidelity of the dynamic simulation for freestyle swimming, we corrected the lower limb motion data using Nakashima’s in-water measurement (Nakashima et al., 2007).

**FIGURE 9:**
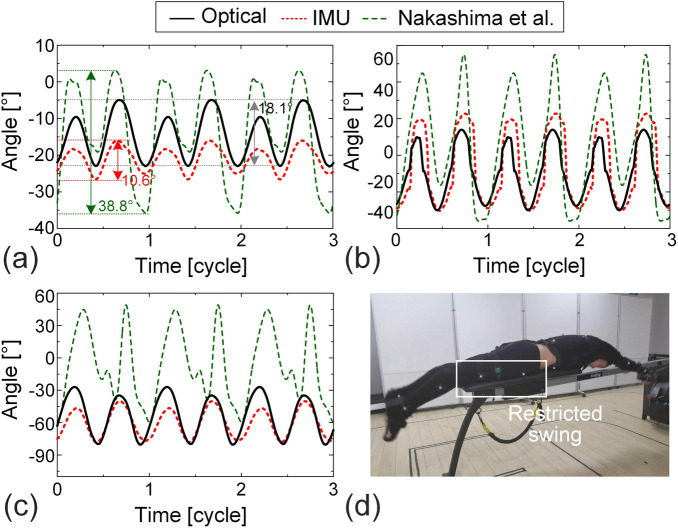
Error analysis and data correction for lower limb motions during butterfly swimming. **(a)** Thigh, **(b)** shank, and **(c)** foot. IMU data, optical motion capture data, and the data reported in Nakashima et al. (2007) are included. **(d)** The snapshot from the test video, showing the restricted motion of the thigh during tests.

The analysis of the butterfly stroke is presented in Figure 9. While the periodic patterns of the optical and IMU data are consistent with those reported by Nakashima et al. (2007), substantial differences are observed in the amplitude and range of motion. For example, the range of thigh motion measured by the IMU and optical system is only 10.6° and 18.1°, respectively, whereas Nakashima’s data shows a significantly larger range of 38.8°, more than double the values recorded in our study (Figure 9a). Similar discrepancies are evident in the shank and foot segments; the measured ranges in our data are less than half of those reported by Nakashima (Figures 9b,c).

These pronounced differences are primarily due to the physical constraints imposed by the onshore swim trainer. Specifically, as shown in Figure 9d, the sliding mat restricts the downward swing of the thighs during the butterfly stroke, thereby limiting the motion of the shank and foot as well. In addition, the absence of buoyancy also affects the lower-limb movements in the butterfly stroke. Such constraints substantially reduce the measured lower-limb motion range and, if uncorrected, can negatively impact the accuracy of dynamic simulation results. As a result, to enhance the realism and reliability of dynamic simulation for butterfly swimming, we corrected the lower-limb motion data using the in-water measurements provided by Nakashima et al. (2007). Specifically, the corrected lower-limb data require time-shifting to align with the original IMU experimental data. Taking the time series of the angle between the thigh and the *x*-axis from the corrected data as a reference, and then time-shifted it so that its minimum value coincided with that of our IMU data. The corrected data of the remaining lower limb segments are also shifted by the same amount.

### 3.2 Dynamic simulation, performance analysis, and motion optimization of human swimming

By integrating wearable IMU-based measurements with onshore swim trainers and employing a multi-rigid-body dynamic model, this study provides an efficient and accessible approach for assessing human swimming performance, optimizing motions, and supporting training interventions. In this section, we demonstrate the effectiveness of the proposed framework—combining onshore motion measurement and dynamic simulation—by first investigating the impact of SF on swimming performance. Through dynamic simulation, we analyze how different stroke frequencies influence key performance metrics. Specifically, the stroke frequencies for breaststroke and butterfly strokes are set at 30, 40, and 50 SPM, while those for freestyle are set at 60, 80, and 100 SPM. The corresponding dynamic simulation animations can be found in the supplementary videos. Following this analysis, we present two illustrative examples highlighting how our methodological framework can be applied to optimize swimming techniques.

#### 3.2.1 Dynamic simulation of breaststroke swimming

**FIGURE 10:**
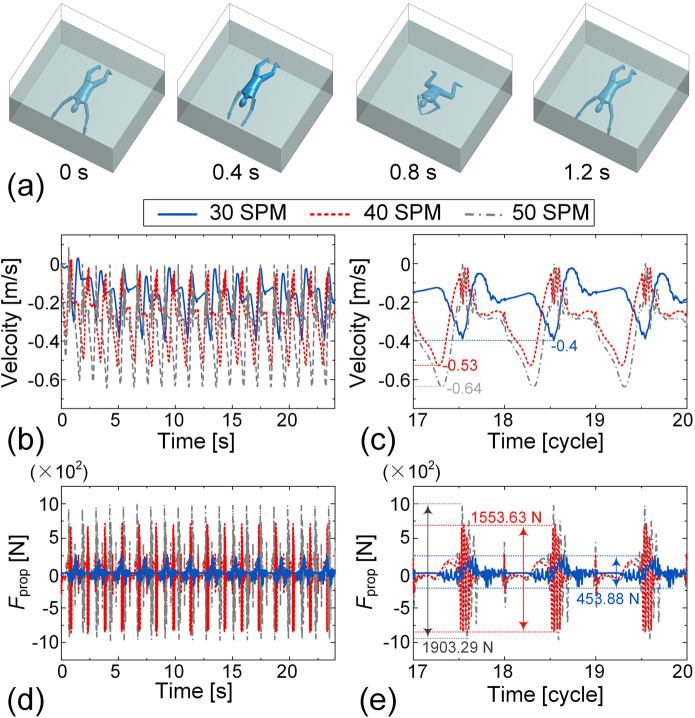
Dynamic simulation of breaststroke swimming at frequencies of 30 SPM, 40 SPM, and 50 SPM. **(a)** Representative snapshots of breaststroke simulation during one cycle at a frequency of 50 SPM. The velocity-time histories over the 24-s interval **(b)** and the final three cycles **(c)** are presented. The time histories of the propulsion force over the 24-s simulation **(d)** and the final three cycles **(e)** are plotted.

For the dynamic simulation of breaststroke, all motion data are derived from IMU measurements, and the motions of the left and right sides of the human body are set to be perfectly symmetric, ensuring unidirectional swimming without lateral deviation. Figure 10a shows representative snapshots of the breaststroke motion during one cycle at a frequency of 50 SPM, as visualized through the dynamic simulation (see Supplementary Video S1 for animation). Figure 10b illustrates the simulated swimming velocity over a 24-s interval for three different stroke frequencies. Since the swimming direction in the simulation is set towards the negative *x*-axis, the velocity values are reported as largely negative. To highlight detailed trends, the velocity-time histories for the final three cycles are enlarged in Figure 10c. The overall temporal profiles of swimming velocity exhibit similar patterns across different stroke frequencies, reflecting the consistent breaststroke technique. As SF increases, swimming velocity also increases. At 50 SPM, the peak speed reaches 0.64 m/s, whereas at 30 SPM, the peak speed is reduced to 0.40 m/s, demonstrating the positive correlation between SF and swimming speed in breaststroke.

Furthermore, we examine the propulsion force $F_{\text{prop}}$ at different stroke frequencies. Figures 10d,e show the time history of the propulsion force over the full 24-s simulation and a magnified view of the final three cycles, respectively, to facilitate detailed comparison. Notably, the period of the PF signal aligns with that of the swimming velocity, reflecting the cyclic nature of the stroke. As SF increases, the peak-to-peak value of $F_{\text{prop}}$ also increases significantly. For example, when the SF rises from 30 SPM to 40 SPM and 50 SPM, the corresponding peak-to-peak force value increases markedly from 453.88 N to 1553.63N and 1903.29 N, respectively. These increases highlight the substantial influence of SF on PF generation in breaststroke and help explain the observed increases in swimming velocity at higher stroke frequencies.

#### 3.2.2 Dynamic simulation of freestyle swimming

**FIGURE 11:**
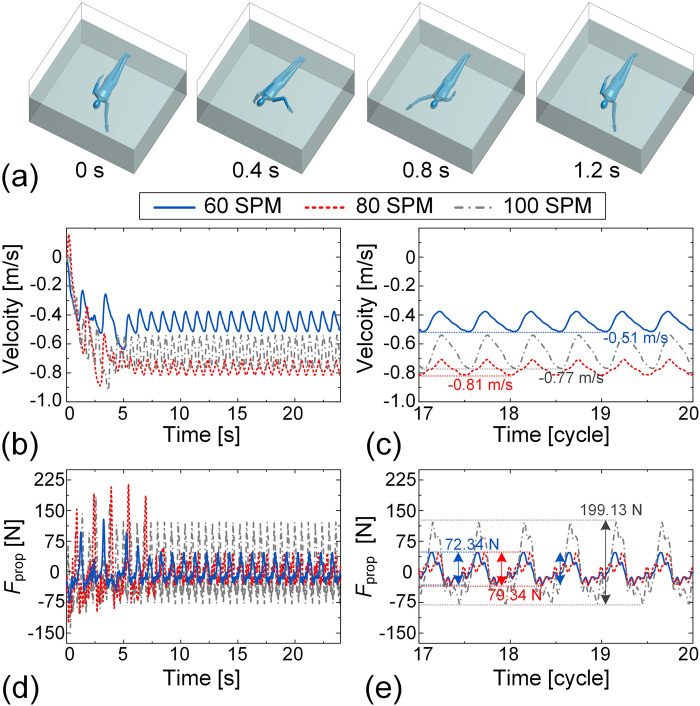
Dynamic simulation of freestyle swimming at frequencies of 60 SPM, 80 SPM, and 100 SPM. **(a)** Representative snapshots of freestyle simulation during one cycle at a frequency of 100 SPM. The velocity-time histories over the 24-s interval **(b)** and the final three cycles **(c)** are presented. The time histories of the propulsion force over the 24-s simulation **(d)** and the final three cycles **(e)** are plotted.

For the dynamic simulation of freestyle, the motions of the left and right sides of the human body are phase synchronized. Motion data for the upper limbs and trunk are derived from IMU measurements, while the motion data for the lower limbs are adopted from Nakashima (2005) and adjusted to match the specified stroke frequencies. Figure 11a presents snapshots of the freestyle motion over one cycle at a frequency of 100 SPM (see Supplementary Video S2 for animation). Unlike breaststroke, the maximum swimming speed in freestyle is achieved at 80 SPM, reaching 0.81 m/s, whereas the velocity at 100 SPM decreases to 0.77 m/s (Figures 11b,c). Despite this, the peak-to-peak PF still exhibits a positive correlation with the SF. Specifically, at 100 SPM, the peak-to-peak values reach 199.13 N, decreasing to 79.34 N and 72.34 N at 80 SPM and 60 SPM, respectively (Figures 11d,e). This discrepancy between PF and swimming velocity can be attributed to differences in upper limb motion patterns across the three stroke frequencies. While the lower limb motion pattern remains consistent across different stroke frequencies, variations in upper limb movement at higher stroke frequencies likely affect propulsion efficiency, ultimately influencing swimming speed.

#### 3.2.3 Dynamic simulation of butterfly swimming

**FIGURE 12:**
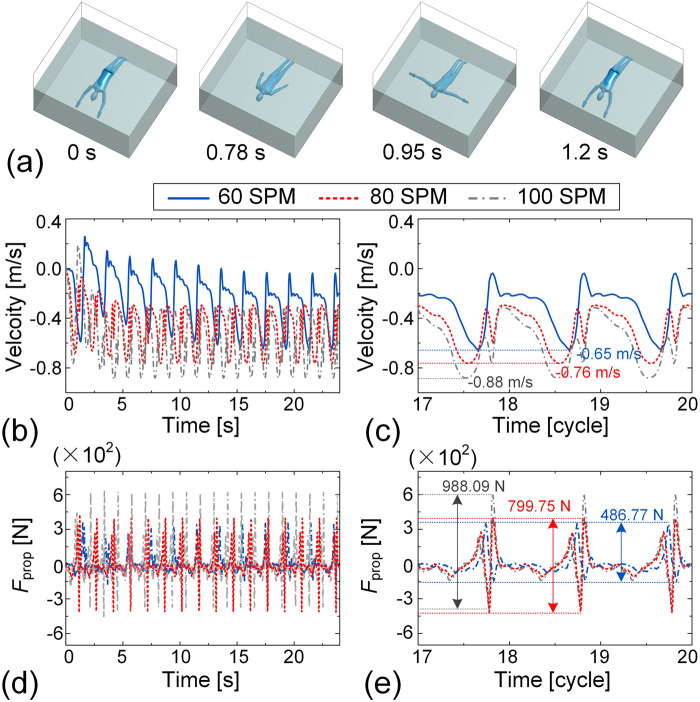
Dynamic simulation of butterfly swimming at frequencies of 30 SPM, 40 SPM, and 50 SPM. **(a)** Representative snapshots of butterfly simulation during one cycle at a frequency of 50 SPM. The velocity-time histories over the 24-s interval **(b)** and the final three cycles **(c)** are presented. The time histories of the propulsion force over the 24-s simulation **(d)** and the final three cycles **(e)** are plotted.

For the dynamic simulation of butterfly, the motions of the left and right sides of the human body are set to be perfectly symmetric. Motion data for the upper limbs and trunk are derived from IMU measurements, while the motion data for the lower limbs are adopted from Nakashima (2005) and adjusted to correspond to the specified stroke frequencies. Figure 12a presents snapshots of the freestyle motion over one cycle at a frequency of 50 SPM (see Supplementary Video S3 for animation). As shown in Figures 12b,c, the swimming velocity in the butterfly exhibits a positive correlation with SF. The highest swimming speed 0.88 m/s is reached at 50 SPM, while it decreases to 0.76 m/s at 40 SPM and 0.65 m/s at 30 SPM. A similar trend is observed in the peak-to-peak PF, which increases with SF. Specifically, the peak-to-peak value of $F_{\text{pro}}$ reaches 988.09 N at 50 SPM, significantly higher than at 40 SPM (799.75 N) and 30 SPM (486.77 N), as illustrated in Figures 12d,e. These consistent trends in swimming velocity and PF underscore the critical role of SF in enhancing swimming performance in butterfly stroke, similar to the findings for breaststroke.

#### 3.2.4 Optimization of swimming stroke

The proposed framework enables the measurement of swimming motion on land and the evaluation of swimming dynamics based on these measured motions, thereby offering a foundation for further optimizing swimming technique. Here, two cases are examined to demonstrate the potential of our approach to guide individualized swimming training and motion optimization.

##### 3.2.4.1 Case 1: Optimizing arm movements of freestyle swimming

**FIGURE 13:**
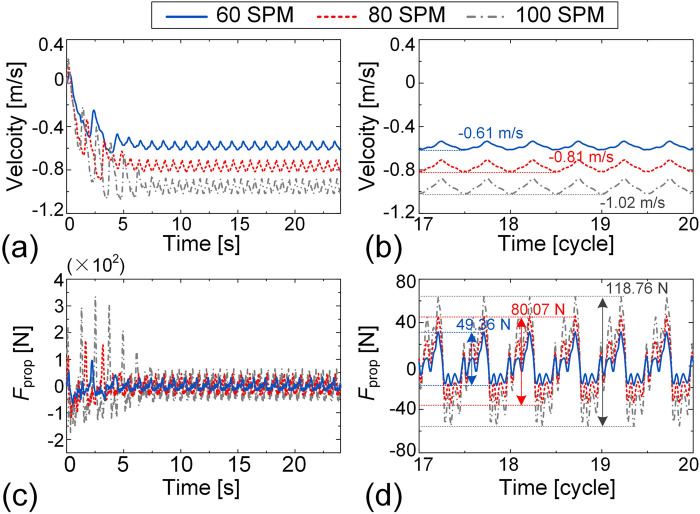
Dynamic simulation results based on optimized arm movements during freestyle swimming at stroke frequencies of 60, 80, and 100 SPM. The time history of the velocity for 0–24 s **(a)** and 18–20 cycles **(b)**. The time history of the propulsion force for 0–24 s **(c)** and 18–20 cycles **(d)**.

Our earlier results in Figure 11 revealed that increasing the freestyle SF from 80 SPM to 100 SPM does not lead to improvement in swimming speed, which we hypothesize may be due to suboptimal arm movement patterns at higher frequencies. To investigate this, we explore whether modifying arm movements can enhance swimming performance. Specifically, we use the arm motion data from the 80 SPM condition as a baseline and adjust the time interval of the time series data to generate signals for 60 SPM and 100 SPM. Using these modified signals as input, we reperform the swimming dynamic simulation (see Supplementary Video S4 for animation). In contrast to the original results in Figure 11, the optimized upper limb movements produced a clear positive correlation between SF and swimming speed (Figure 13). As the SF increases from 60 SPM to 80 SPM and 100 SPM, the maximum swimming speed rises from 0.61 m/s to 0.81 m/s and 1.02 m/s, respectively (Figures 13a,b). Similarly, the peak-to-peak propulsion force shares the same trend, increasing from 49.36 N at 60 SPM to 80.07 N at 80 SPM, and reaching 118.76 N at 100 SPM (Figures 13c,d). These findings confirm that the lack of speed improvement at 100 SPM in Figure 11 was due to suboptimal arm movements, and demonstrate the potential of our framework for swimming motion optimization and training guidance.

##### 3.2.4.2 Case 2: Optimizing trunk movements of butterfly swimming

**FIGURE 14:**
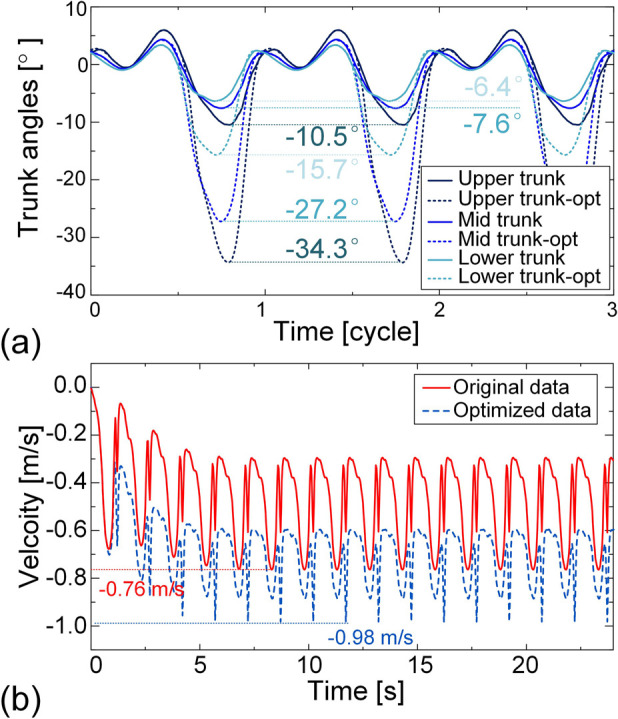
Optimization of butterfly stroke movements at a SF of 40 SPM. **(a)** The optimized joint angle of the upper trunk, middle trunk, and lower trunk. **(b)** The time history of the velocity based on original data and optimized data.

Figure 12 reveals that during butterfly stroke on the onshore swim trainer, the swimmer is unable to perform a downward trunk swing—a motion that is both common and critical in actual water-based butterfly stroke. This limitation likely compromises swimming performance. To evaluate the impact of trunk movement on performance, we examine whether optimizing the trunk motion can improve butterfly stroke efficiency. We select the 40 SPM butterfly as a representative example. To restore realistic trunk motion, the trunk kinematic data reported by Nakashima (2005) is adopted to modify the IMU-measured trunk signals, ensuring that the downward swing is properly reflected. Specifically, the lower bound angles of the upper trunk, middle trunk, and lower trunk are adjusted from −10.5°, −7.6°, and −6.4° to −34.3°, −27.2°, and −15.7°, respectively (Figure 14a). Using these optimized signals, a new dynamic simulation is conducted (see Supplementary Video S5 for animation). The results reveal a substantial improvement in butterfly swimming performance, with the maximum speed increasing by 29.0%, from −0.76 m/s to −0.98 m/s (Figure 14b). This outcome underscores the significant role of trunk dynamics in butterfly stroke and further manifests the feasibility and potential of our proposed framework for guiding motion optimization.

## 4 Discussion

### 4.1 Limitations and future research directions

1. Although the swimming motion performed on the onshore swim trainer ensures a horizontal body posture and simulates swimming resistance, it cannot fully reproduce the buoyancy effects experienced during real swimming in water, and the muscle activation patterns during land-based swimming differ substantially from those in real swimming, which may easily lead to deformation of the swimming motion. Therefore, the land-based swimming measurement data presented in this research, as well as the corresponding simulation and optimization results, primarily demonstrate the feasibility and validity of this methodological framework. This enables it to serve as a supplementary tool or preliminary screening method to guide swimming training and swimming robot development.
2. During the experiments, the sliding mat of the onshore swim trainer imposed significant constraints on the motion of the lower limbs, causing severe compression of the thigh muscles and resulting in soft-tissue artifact issues during measurement, which led to substantial discrepancies between the calculated lower-limb (i.e., thigh, shank, and foot) angles and those obtained from optical motion capture. This is the primary reason for the significant differences observed in indexes such as the Absolute Agreement Intraclass Correlation Coefficient (ICC). In existing research, Bouvier et al. (2015) reduced soft tissue artefact by mounting IMUs on rigid plates equipped with clusters of optical markers, which could be considered in our future studies.
3. In the land-based swimming test data, we indeed observed differences between the left and right side motions as well as variations across different periods; however, to ensure simulation convergence, the data are artificially symmetrized, which represents one of the limitations of this study. In reality, during real swimming in water, humans autonomously adjust their force output to maintain swimming direction, whereas on the onshore swim trainer, such adjustments are not possible. Therefore, in future studies, if real swimming data can be collected, the mechanism of autonomous directional adjustment could be explored based on a multi-rigid-body dynamics model.
4. In this study, only one participant is used to verify the proposed methodological framework; therefore, the conclusions drawn here should be regarded only as a proof of concept. Applying this framework to participants with different swimming abilities for testing, analysis, and optimization is generally feasible; however, further studies involving more participants of different skill levels and real swimming scenarios are still required.
5. In the multibody dynamics model we established, the shape, density, and center of mass of the human body segments are simplified. The dynamic model does not directly employ joint actuation but instead uses human motion signals as inputs. Fluid dynamic effects such as passive drag, active drag, and buoyancy are considered using a simplified hydrodynamic model, while the influences of waves and vortices are not taken into account. However, these factors may all affect the accuracy of the simulation. Therefore, future work should focus on refining the hydrodynamic model.
6. The optimization of swimming motions in this study also involves a certain degree of subjectivity, and whether the optimized movements can actually be performed by the human body still requires guidance from biomechanics experts and professional coaches.

It should be noted that although the swimming analysis framework proposed in this work has the above limitations, they do not affect the verification of its feasibility. Once the feasibility is confirmed, the significance of this framework can be ensured when comprehensive human swimming data collected in real aquatic environments are used. Therefore, although the measurement data used in this study are not entirely comprehensive, this does not affect the validation of the proposed framework’s effectiveness.

### 4.2 Application prospects

1. For sports training, coaches and athletes can not only obtain complete motion data via IMUs but, more importantly, can use these data as the basis to visualize simulated movement animations through the multibody dynamics model. This provides a clearer understanding of swimming techniques. Furthermore, it can serve as a foundation to optimize specific body segment motions of the athlete and to preliminarily assess the potential improvements in performance resulting from motion optimization. Both these capabilities are beyond the scope of a traditional motion capture system.
2. For swimming robot development, full-body motion optimization can be performed based on human motion data. Then, the robot can be abstracted as a multibody dynamics model to evaluate the effects of the optimized motions on its functional performance, enabling motion planning for individual joints.

## 5 Conclusion

Onshore measurement and analysis of human swimming face several challenges, including accurately capturing motion data in horizontal swimming postures, assessing signal quality across all body segments, and evaluating swimming dynamics based on measured motion data. In this study, we propose an integrated framework that combines wearable IMUs, onshore swim trainer, and a multi-rigid-body dynamic model for comprehensive swimming motion analysis and performance assessment. Experimental results demonstrate that IMU measured kinematic data are highly consistent with optical motion capture data for most body segments, with Spearman correlation coefficients exceeding 0.75, ICC values mostly above 0.75, and NRMSE values below 25%. However, due to physical constraints imposed by the onshore swim trainer—particularly limiting the downward swing of the lower limbs and trunk—the measured signals for these segments deviate from typical in-water swimming motions.

We established a multi-rigid-body Newton-Euler dynamic model that incorporates fluid forces to simulate swimming dynamics based on the measured kinematic data. Simulation results show that the SF significantly affects swimming speed and propulsion force in breaststroke, freestyle, and butterfly strokes. Moreover, two case studies—optimizing arm movement in freestyle and trunk movement in butterfly—demonstrate that the proposed framework can effectively support swimming motion optimization and performance enhancement.

The swimming motion analysis framework proposed in this work could serve as a supplementary tool and preliminary screening method for guiding swimming training and swimming robot development. Future work includes involving participants of varying skill levels, re-evaluating swimming measurements in real aquatic environments, and integrating biomechanics for analysis and optimization to guide robot design.

## Data Availability

The raw data supporting the conclusions of this article will be made available by the authors, without undue reservation.
