# Supplementary material for: Onshore human swimming motion measurement and dynamic analysis using wearable inertial sensors
Source: Front Bioeng Biotechnol. 2026 May 7;14:1791337. doi: 10.3389/fbioe.2026.1791337 (PMC13189736; doi:10.3389/fbioe.2026.1791337)
Supplement: Supplementary file 2 [file Supplementaryfile1.docx]

Supplementary Material for

Onshore Human Swimming Motion Measurement and Dynamic Analysis

Using Wearable Inertial Sensors

# Supplementary Material S1: The detailed implementation of the IMUs (Perception Neuron Studio)

**1.1 The specifications of Perception Neuron Studio**

According to the documentation provided by the Perception Neuron Studio (PNS) manufacturer, the detailed specifications of the internal sensors are as follows: gyroscope measurement range: ±2000 dps; accelerometer measurement range ±32 g. Regarding the magnetometer, the manufacturer does not publicly disclose the specific hardware measurement range in their technical datasheets, as the system functions as a closed-source commercial unit. However, it should be noted that the magnetometer integrated in the PNS continuously provides the absolute direction of the Earth’s magnetic field during testing, thereby correcting drift errors. In addition, its accompanying software, Axis Studio, incorporates an anti-magnetic interference function. If the testing environment is extremely unfavorable, the system dynamically reduces its reliance on the magnetometer and instead utilizes stronger human kinematic constraints to stabilize the skeletal posture and prevent model collapse.

**1.2 IMU placement on the subject**

To minimize soft tissue artifacts, the sensor-to-segment mounting procedure is standardized by firmly attaching the IMUs to specific anatomical regions away from prominent muscle bellies as shown in **Table S1**.

TABLE S1

Position for wearing the PNS sensor

| **Segment** | **Explanation** |
| --- | --- |
| Head | The strap is placed on the upper part of the head, with its lower edge passing just above the superciliary arch and the auricles. The sensor is located in the center of the forehead. |
| Upper back | The strap passes under the armpits. The sensor is located behind the sixth thoracic vertebra. |
| Shoulder (left/right) | The sensor is located above the spine of the scapula, at the medial border of the scapula. |
| Upper arm (left/right) | The strap is placed over the humerus. The sensor is located on the exact lateral side of the upper arm, centered at the deltoid tuberosity. |
| Forearm (left/right) | The lower edge of the strap is positioned just above the ulnar tuberosity. The sensor is located at the anterior 1/3 of the forearm. |
| Hand (left/right) | The strap passes through the purlicue (the web between the thumb and index finger). The sensor is located in the center of the back of the hand. |
| Lower Back | The lower edge of the strap passes along the left and right iliac crests. The sensor is aligned horizontally with the third lumbar vertebra. |
| Thigh (left/right) | The strap is placed at the center of the longitudinal axis of the thigh. The sensor is located at the iliotibial band. |
| Shank (left/right) | The strap is placed at the lower-middle part of the shank, with its upper edge passing below the bulge of the posterior calf muscles. The sensor is located on the anteromedial surface of the tibia. |
| Foot (left/right) | The strap passes through the center of the longitudinal axis of the foot arch. The sensor is located directly above the foot arch. |

**1.3 The pre-defined human skeletal model of IMU**

The pre-defined human skeletal model is not a set of independent and unconstrained body segments in the global space. Instead, it adopts a top-down hierarchical rigid-body kinematic chain that conforms to the Biovision Hierarchy (BVH) standard. For our analysis, the Hip is defined as the global root node, and the lower-limb segments are hierarchically connected in sequence. Before testing, the system calibrated the lengths of each body segment in the model according to the actual geometric parameters of the subject.

TABLE S2

The detailed information of the pre-defined human skeletal model

| **Segment** | **Motion** | **Explanation** | **Description** |
| --- | --- | --- | --- |
| Head | Flexion/Extension | Looking down is flexion (+), looking up is extension (-) | Rotation around the frontal axis within the sagittal plane |
|  | Rotation | Left rotation is positive (+), right rotation is negative (-) | Rotation around the vertical axis within the transverse plane |
|  | Lateral Flexion | Left lateral flexion is positive (+), right is negative (-) | Rotation around the sagittal axis within the frontal plane |
| Trunk | Flexion/Extension | Bending forward is flexion (+), bending backward is extension (-) | Rotation around the frontal axis within the sagittal plane |
|  | Rotation | Left rotation is positive (+), right is negative (-) | Rotation around the vertical axis within the transverse plane |
|  | Lateral Flexion | Left lateral flexion is positive (+), right is negative (-) | Rotation around the sagittal axis within the frontal plane |
| Shoulder | Flexion/Extension | Raising the upper arm forward is flexion (+), backward is extension (-) | Rotation around the frontal axis within the sagittal plane |
|  | Internal/External Rotation | Right arm: Counter-clockwise from proximal to distal is internal rotation (+), otherwise external (-); Left arm: Opposite to right | Rotation around the vertical axis within the transverse plane |
|  | Adduction/Abduction | Both arms: Moving inward is adduction (+), outward is abduction (-) | Rotation around the sagittal axis within the frontal plane |
| Elbow | Flexion/Extension | Bending the elbow forward is flexion (+), extending backward is extension (-) | Rotation around the frontal axis within the sagittal plane |
|  | Adduction/Abduction | Both arms: Moving inward is adduction (+), outward is abduction (-) | Rotation around the sagittal axis within the frontal plane |
| Forearm | Pronation/Supination | Right arm: Counter-clockwise from proximal to distal is pronation (+), otherwise supination (-); Left arm: Same logic | Rotation around the vertical axis within the transverse plane |
| Wrist | Flexion/Extension | Bending palm toward forearm is flexion (+), extending back is extension (-) | Rotation around the frontal axis within the sagittal plane |
|  | Internal/External Rotation | Right hand: Counter-clockwise from proximal to distal is internal rotation (+), otherwise external (-); Left hand: Opposite | Rotation around the vertical axis within the transverse plane |
|  | Ulnar/Radial Deviation | Ulnar deviation: Palm moves toward little finger (+); Radial deviation: Palm moves toward thumb (-) | Rotation around the sagittal axis within the frontal plane |
| Hip | Flexion/Extension | Raising thigh forward is flexion (+), backward is extension (-) | Rotation around the frontal axis within the sagittal plane |
|  | Internal/External Rotation | Right thigh: Counter-clockwise from proximal to distal is internal rotation (+), otherwise external (-); Left thigh: Opposite | Rotation around the vertical axis within the transverse plane |
|  | Adduction/Abduction | Both thighs: Moving inward is adduction (+), outward is abduction (-) | Rotation around the sagittal axis within the frontal plane |
| Knee | Flexion/Extension | Bending lower leg backward is flexion (+), otherwise extension (-) | Rotation around the frontal axis within the sagittal plane |
|  | Internal/External Rotation | Right lower leg: Counter-clockwise from proximal to distal is internal rotation (+), otherwise external (-); Left lower leg: Opposite | Rotation around the vertical axis within the transverse plane |
|  | Adduction/Abduction | Both lower legs: Moving inward is adduction (+), outward is abduction (-) | Rotation around the sagittal axis within the frontal plane |
| Ankle | Flexion/Extension | Pointing toes downward (plantar flexion) is flexion (+), otherwise extension (-) | Rotation around the frontal axis within the sagittal plane |
|  | Internal/External Rotation | Right foot: Counter-clockwise from proximal to distal is internal rotation (+), otherwise external (-); Left foot: Opposite | Rotation around the vertical axis within the transverse plane |
|  | Inversion/Eversion | Inversion: Sole turns inward along the ground direction (+); Eversion: Opposite (-) | Rotation around the sagittal axis within the frontal plane |

The segment connections in the hierarchical model operate as 3-degree-of-freedom (3-DoF) spherical joints to capture full three-dimensional rotations. However, they are strictly constrained during the mathematical solving process. The proprietary Axis Studio computational algorithm explicitly incorporates human kinematic constraints (as shown in the **Table S2**) as well as environmental contact constraints (e.g., foot–ground contact) during the solving process. This optimization framework applies a joint-constraint penalty mechanism to prevent non-physiological joint dislocations (such as unnatural hip separation) and enforces anatomical joint range-of-motion (ROM) limits during dynamic tasks.

The complete computational pipeline for estimating the Euler angle is as follows: 1) the raw IMU data are processed using an AHRS algorithm to estimate the global spatial orientation of each sensor node; 2) the sensors are aligned with the body segments using a standardized static calibration procedure (T-pose/A-pose); 3) the algorithm reconstructs joint kinematics along the hierarchical kinematic chain based on joint constraints and environmental constraints, ultimately obtaining the Euler angle data of each body segment.

**1.4 Details of PNS data processing**

According to the manufacturer’s documentation, the PNS system utilizes full 9-axis IMU nodes. The sensor fusion framework continuously incorporates both accelerometer data (to establish a reliable gravity vector for correcting Pitch and Roll drift) and magnetometer data (to provide an absolute heading reference for correcting Yaw drift), alongside the high-frequency angular velocity from the gyroscope.

The raw data are processed using the system’s built-in Attitude and Heading Reference System (AHRS). While the exact source code is closed as a commercial product, the manufacturer specifies that it employs an advanced multi-sensor fusion filtering algorithm (Extended Kalman Filter method) to optimally fuse the 9-axis data and reject environmental noise and magnetic disturbances.

# Supplementary Material S2: Comprehensive results of validity, reliability, and accuracy evaluation of all body segments in three strokes

TABLE S3

Validity, reliability and accuracy of the three swimming strokes

|  | **Index** | **Breaststroke** | **Freestyle** | **Butterfly** |
| --- | --- | --- | --- | --- |
| **Angle between head and x-axis** | *Spearman r* | 0.816 | 0.839 | 0.824 |
|  | Linear regression function | *y*=0.834*x*-4.643 | *y*=0.878*x*-3.266 | *y*=0.759*x*-10.997 |
|  | *R^2^* | 0.744 | 0.712 | 0.821 |
|  | *ICC* (1,1) | 0.794 | 0.715 | 0.665 |
|  | *NRMSE (%)* | 30.748 | 14.619 | 26.024 |
| **0Angle between right upper arm and x-axis** | *Spearman r* | 0.961 | 0.982 | 0.992 |
|  | Linear regression function | *y*=0.884*x*+14.987 | *y*=0.929*x*+8.188 | *y*=0.950*x*+5.079 |
|  | *R^2^* | 0.916 | 0.967 | 0.990 |
|  | *ICC* (1,1) | 0.948 | 0.979 | 0.994 |
|  | *NRMSE (%)* | 10.567 | 6.537 | 3.834 |
| **Angle between right upper arm and y-axis** | *Spearman r* | 0.859 | 0.989 | 0.995 |
|  | Linear regression function | *y*=0.780*x*+33.065 | *y*=1.034*x*+0.836 | *y*=1.004*x*+3.087 |
|  | *R^2^* | 0.715 | 0.981 | 0.988 |
|  | *ICC* (1,1) | 0.814 | 0.981 | 0.982 |
|  | *NRMSE (%)* | 16.861 | 6.061 | 5.956 |
| **Angle between right upper arm and z-axis** | *Spearman r* | 0.912 | 0.552 | 0.944 |
|  | Linear regression function | *y*=0.964*x*+0.148 | *y*=0.914*x*+7.580 | *y*=1.029*x*-4.853 |
|  | *R^2^* | 0.931 | 0.740 | 0.969 |
|  | *ICC* (1,1) | 0.952 | 0.851 | 0.981 |
|  | *NRMSE (%)* | 8.982 | 14.449 | 4.836 |
| **Angle between left upper arm and x-axis** | *Spearman r* | 0.959 | 0.995 | 0.993 |
|  | Linear regression function | *y*=0.887*x*+11.335 | *y*=0.865*x*+11.439 | *y*=0.871*x*+12.270 |
|  | *R^2^* | 0.928 | 0.991 | 0.989 |
|  | *ICC* (1,1) | 0.960 | 0.985 | 0.985 |
|  | *NRMSE (%)* | 9.454 | 5.766 | 6.236 |
| **Angle between left upper arm and y-axis** | *Spearman r* | 0.985 | 0.927 | 0.933 |
|  | Linear regression function | *y*=0.992*x*-4.893 | *y*=0.971*x*+0.992 | *y*=0.947*x*+4.373 |
|  | *R^2^* | 0.924 | 0.875 | 0.806 |
|  | *ICC* (1,1) | 0.921 | 0.933 | 0.892 |
|  | *NRMSE (%)* | 10.181 | 11.401 | 14.117 |
| **Angle between left upper arm and z-axis** | *Spearman r* | 0.778 | 0.983 | 0.798 |
|  | Linear regression function | *y*=0.784*x*-14.905 | *y*=1.325*x*-18.832 | *y*=0.743*x*+15.838 |
|  | *R^2^* | 0.671 | 0.952 | 0.668 |
|  | *ICC* (1,1) | 0.816 | 0.926 | 0.810 |
|  | *NRMSE (%)* | 16.634 | 9.219 | 15.762 |
| **Right Elbow** | *Spearman r* | 0.963 | 0.945 | 0.888 |
|  | Linear regression function | *y*=0.756*x*+10.504 | *y*=0.844*x*-6.138 | *y*=0.964*x*+1.266 |
|  | *R^2^* | 0.938 | 0.955 | 0.889 |
|  | *ICC* (1,1) | 0.913 | 0.957 | 0.942 |
|  | *NRMSE (%)* | 13.099 | 8.912 | 8.451 |
| **Left Elbow** | *Spearman r* | 0.929 | 0.947 | 0.577 |
|  | Linear regression function | *y*=0.692*x*+16.051 | *y*=0.766*x*-9.833 | *y*=0.658*x*+16.845 |
|  | *R^2^* | 0.956 | 0.978 | 0.799 |
|  | *ICC* (1,1) | 0.901 | 0.917 | 0.846 |
|  | *NRMSE (%)* | 15.324 | 10.545 | 17.333 |
| **Angle between right thigh and x-axis** | *Spearman r* | 0.875 | 0.615 | 0.915 |
|  | Linear regression function | *y*=1.299*x*+11.651 | *y*=1.371*x*+9.892 | *y*=1.527*x*+15.339 |
|  | *R^2^* | 0.856 | 0.391 | 0.814 |
|  | *ICC* (1,1) | 0.840 | 0.119 | 0.420 |
|  | *NRMSE (%)* | 12.754 | 29.973 | 27.074 |
| **Angle between right thigh and y-axis** | *Spearman r* | 0.755 | 0.751 | 0.951 |
|  | Linear regression function | *y*=0.823*x*+13.379 | *y*=1.568*x*-67.493 | *y*=1.395*x*-46.104 |
|  | *R^2^* | 0.640 | 0.550 | 0.889 |
|  | *ICC* (1,1) | 0.166 | 0.039 | 0.682 |
|  | *NRMSE (%)* | 25.010 | 38.467 | 20.874 |
| **Angle between right thigh and z-axis** | *Spearman r* | 0.962 | 0.506 | 0.214 |
|  | Linear regression function | *y*=1.210*x*-18.759 | *y*=0.349*x*+57.379 | *y*=0.116*x*+75.628 |
|  | *R^2^* | 0.925 | 0.267 | 0.036 |
|  | *ICC* (1,1) | 0.929 | 0.479 | 0.038 |
|  | *NRMSE (%)* | 10.299 | 32.461 | 50.121 |
| **Angle between left thigh and x-axis** | *Spearman r* | 0.509 | 0.565 | 0.975 |
|  | Linear regression function | *y*=0.725*x*-1.478 | *y*=1.305*x*+8.726 | *y*=1.186*x*+5.592 |
|  | *R^2^* | 0.651 | 0.328 | 0.929 |
|  | *ICC* (1,1) | 0.693 | 0.126 | 0.808 |
|  | *NRMSE (%)* | 19.465 | 26.512 | 20.758 |
| **Angle between left thigh and y-axis** | *Spearman r* | 0.678 | 0.525 | 0.979 |
|  | Linear regression function | *y*=1.047*x*-10.207 | *y*=0.971*x*-3.354 | *y*=1.523*x*-58.630 |
|  | *R^2^* | 0.571 | 0.290 | 0.942 |
|  | *ICC* (1,1) | 0.038 | -0.428 | 0.688 |
|  | *NRMSE (%)* | 23.988 | 42.237 | 19.723 |
| **Angle between left thigh and z-axis** | *Spearman r* | 0.956 | 0.714 | 0.739 |
|  | Linear regression function | *y*=1.296*x*-24.859 | *y*=0.580*x*+40.766 | *y*=0.456*x*+52.119 |
|  | *R^2^* | 0.948 | 0.533 | 0.556 |
|  | *ICC* (1,1) | 0.935 | 0.525 | 0.522 |
|  | *NRMSE (%)* | 9.485 | 24.849 | 30.161 |
| **Right knee** | *Spearman r* | 0.789 | 0.544 | 0.347 |
|  | Linear regression function | *y*=1.046*x*-12.299 | *y*=0.228*x*+4.582 | *y*=0.193*x*+5.429 |
|  | *R^2^* | 0.957 | 0.370 | 0.253 |
|  | *ICC* (1,1) | 0.943 | 0.399 | 0.326 |
|  | *NRMSE (%)* | 10.431 | 41.383 | 63.938 |
| **Left knee** | *Spearman r* | 0.739 | 0.697 | 0.789 |
|  | Linear regression function | *y*=1.016*x*-8.716 | *y*=0.555*x*+5.977 | *y*=0.426*x*+5.429 |
|  | *R^2^* | 0.973 | 0.606 | 0.814 |
|  | *ICC* (1,1) | 0.963 | 0.720 | 0.639 |
|  | *NRMSE (%)* | 8.586 | 22.485 | 41.223 |
| **Angle between right shank and x-axis** | *Spearman r* | 0.853 | 0.968 | 0.945 |
|  | Linear regression function | *y*=0.907*x*-0.617 | *y*=0.707*x*-3.960 | *y*=0.657*x*-6.409 |
|  | *R^2^* | 0.931 | 0.901 | 0.863 |
|  | *ICC* (1,1) | 0.963 | 0.893 | 0.855 |
|  | NRMSE (%) | 9.138 | 15.651 | 20.799 |
| **Angle between left shank and x-axis** | *Spearman r* | 0.859 | 0.965 | 0.994 |
|  | Linear regression function | *y*=0.938*x*-0.733 | *y*=0.842*x*-3.877 | *y*=0.934*x*-2.579 |
|  | *R^2^* | 0.923 | 0.933 | 0.986 |
|  | *ICC* (1,1) | 0.960 | 0.913 | 0.980 |
|  | NRMSE (%) | 9.223 | 13.359 | 7.128 |
| **Angle between right foot and x-axis** | *Spearman r* | 0.396 | 0.982 | 0.961 |
|  | Linear regression function | *y*=1.037*x*+5.855 | *y*=0.873*x*-2.982 | *y*=1.072*x*+9.379 |
|  | *R^2^* | 0.416 | 0.953 | 0.913 |
|  | *ICC* (1,1) | 0.572 | 0.951 | 0.903 |
|  | *NRMSE (%)* | 17.721 | 8.745 | 10.683 |
| **Angle between left foot and x-axis** | *Spearman r* | 0.788 | 0.988 | 0.921 |
|  | Linear regression function | *y*=0.729*x*-18.151 | *y*=0.990*x*-6.040 | *y*=0.810*x*-15.127 |
|  | *R^2^* | 0.531 | 0.977 | 0.856 |
|  | *ICC* (1,1) | 0.723 | 0.952 | 0.894 |
|  | *NRMSE (%)* | 13.134 | 8.728 | 13.427 |

# Supplementary Videos: Dynamic simulation animations of the three strokes at different stroke frequencies

Supplementary Video S1: The Dynamic Simulation Animation of Breaststroke

Supplementary Video S2: The Dynamic Simulation Animation of Freestyle

Supplementary Video S3: The Dynamic Simulation Animation of Butterfly

Supplementary Video S4: The Dynamic Simulation Animation of Optimized Freestyle

Supplementary Video S5: The Dynamic Simulation Animation of Optimized Butterfly
